# Therapeutic Use of Cannabis Derivatives and Their Analogs for Autism Spectrum Disorder: A Systematic Review

**DOI:** 10.1002/jcph.70068

**Published:** 2025-07-02

**Authors:** Rachel Riera, Isabela Porto de Toledo, Cecília Menezes Farinasso, Rafael Leite Pacheco, Roberta Borges Silva, Verônica Colpani, Ana Luiza Cabrera Martimbianco, Camila Monteiro Cruz, Patrícia do Carmo Silva Parreira, Carolina de Oliveira Cruz Latorraca

**Affiliations:** ^1^ Hospital Sírio‐Libanês (HSL) São Paulo SP Brazil; ^2^ Escola Paulista de Medicina Universidade Federal de São Paulo (Unifesp) São Paulo SP Brazil; ^3^ Centro Universitário São Camilo (CUSC) São Paulo SP Brazil; ^4^ Universidade Metropolitana de Santos (Unimes) Santos SP Brazil

**Keywords:** autism spectrum disorders, autistic disorder, cannabidiol, cannabis, medical marijuana, systematic review

## Abstract

Autism spectrum disorders are characterized by some difficulties with social interactions and communication, atypical patterns of behavior, and unusual reactions to emotions. Studies have found promising results regarding the effects of cannabis on autism. We conducted a systematic review of randomized clinical trials on the effects of cannabis derivatives and their analogs for autism. This review was developed according to the Cochrane Handbook for Systematic Reviews of Interventions and reported according to PRISMA 2020. The protocol was prospectively published in the PROSPERO database (CRD42023468300). We included randomized controlled trials with autism‐diagnosed participants treated with any cannabis derivate or its analogs for therapeutic purposes. Two reviewers assessed titles and abstracts independently and potentially eligible full texts were assessed to confirm eligibility. After that, they extracted data using a standardized worksheet. Searches retrieved 1264 references, only 11 RCTs were included, four with available results for children/adolescents with autism. Five different cannabis presentations were tested. One trial pointed that cannabis may improve global assessment symptoms, but for other outcomes results were uncertain. No included study assessed quality of life. The certainty of evidence ranged from very low to low certainty for the assessed outcomes. Cannabis whole plant extract may improve global assessment symptoms, but the different cannabis presentations, outcome assessments and very low certainty of evidence from the included studies make it difficult to draw conclusions about cannabis for people with autism. This scenario of uncertainties impacts directly clinical practice and decision making.

## Introduction

Autism spectrum disorders (ASD) are a diverse group of conditions characterized by some degree of difficulties with social interactions and communication, atypical patterns of behavior and activities, and unusual reactions to emotions.^1^ Comorbidities and levels of intellectual impairment may vary. ASD prevalence worldwide is estimated to be 1 in 100 children,^1^ considering that boys receive an ASD diagnosis almost three times more frequently than girls.^2^, ^3^

Although there is no single known cause for the onset of this lifelong condition, several treatments have been developed, from pharmacological to behavioral therapy. For instance, the Applied Behavioral Analysis (ABA) and the Early Start Denver Model (ESDM) are high‐intensity behavioral interventions that can be employed soon after the diagnosis is defined, either at home or at school.^4^ Another strategy, the Treatment and Education of Autistic and Communication Related Handicapped Children (TEACCH), is an educational intervention that aims to provide independence.^5^ Therapeutic strategies should be individualized, periodically reassessed for effectiveness, and adjusted as the needs of the person with ASD evolve.^6^

However, there are cases in which it is necessary to integrate pharmacological treatments depending on the type and severity of the symptoms, such as anxiolytics, antidepressants, stimulants, or antipsychotics.^4^, ^7^ Similarly to non‐pharmacological interventions, there is no single known medication that is sufficient to manage all ASD symptoms, which leads to a continuous search for new alternatives.

There has been investigation on the therapeutic effects of cannabis derivatives and their analogs for clinical conditions such as dementia, epilepsy, fibromyalgia, HIV/AIDS, nausea and vomiting related to chemotherapy in adults and children, schizophrenia, and Tourette's syndrome.^8^, ^9^, ^10^, ^11^, ^12^, ^13^, ^14^ Studies have hypothesized that cannabis could improve symptoms in general for people with ASD, acting on brain function, probably modulating neuronal plasticity, social responses, nociception, and cognition.^15^ One study found that dronabinol may improve aggression and stereotypical behavior.^16^ Another observed that cannabis seems to be well tolerated, safe, and effective in relieving symptoms such as episodes of aggressive behavior.^17^ Cannabis compounds act mainly by interacting with the endocannabinoid system, which is mainly made up of CB1 and CB2 receptors. THC, which is responsible for the cannabis psychoactive effects, may influence memory, movement, appetite, and sense perception.^8^, ^9^, ^10^

Therefore, for guiding the clinical decision, informing public health policies, and supporting further research, a systematic review mapping, critically appraising, and synthesizing the best available evidence about the effects (benefits and harms) of cannabis derivatives and their analogs for ASD is necessary.

## Methods

### Design and Setting

This study is a systematic review of randomized controlled trials (RCTs). The review was carried out in the Health Technology Assessment Center at Hospital Sírio‐Libanês, São Paulo, Brazil. The protocol was prospectively registered in the PROSPERO database (CRD42023468300, available from: https://www.crd.york.ac.uk/prospero/display_record.php?RecordID = 468300). We planned and developed the review according to the Cochrane Handbook for Systematic Reviews of Interventions^18^ and reported according to the Preferred Reporting Items for Systematic Review and Meta‐Analysis (PRISMA 2020) statement.^19^

### Eligibility Criteria

We derived the eligibility criteria from the components of our PICOS question (P: population; I: intervention; C: comparator; O: outcomes of interest; S: study design).

#### Population

Participants diagnosed with ASD at any age, severity of symptoms, time from onset, clinical characteristics, and associated or not to other conditions or neurodevelopmental disorders including (but not limited to) attention deficit hyperactivity disorder. Studies including participants with ASD as a subgroup of a wide population of interest would be considered only if the authors presented subgroup analysis separately for them.

#### Intervention

Any cannabis derivative (including whole plant or extract) or its analogs when used for therapeutic purposes, without restrictions of regimen, dose, or route of administration. Co‐interventions were allowed as long as both the intervention and the comparison group received them. For the control groups, any other active pharmacological or non‐pharmacological intervention, placebo/sham, or no intervention were eligible.

#### Study Design

We only included RCTs, with cluster or individual randomization, since it is recognized as the most appropriate primary study design to assess the effect of a healthcare intervention. Cross‐over trials were eligible, however, only the first phase was considered for analysis. Although other types of comparative primary studies may show some effect of the intervention of interest, biases and other factors can strongly impact the results, reinforcing the importance of prioritizing only clinical trials.

The primary outcomes of this systematic review were:

‐ Global assessment of symptoms using the Autism Treatment Evaluation Checklist (ATEC),^20^ Behavioral Summarized Evaluation (BSE),^21^ or any other validated and specific tool for clinical evaluation.
‐ Severity of autism symptoms by any validated tool.
‐ Serious adverse events assessed by the frequency of participants who experienced at least one serious adverse event as defined by the FDA^22^ or by the study authors.

The secondary outcomes of this systematic review were quality of life (measured by any validated tool); any adverse events (expressed by the frequency of people who experienced at least one adverse event); and adaptive behavior (as assessed by any validated tool).

The outcomes were considered when measured at any time point. However, only similar time points were gathered in a meta‐analysis: short term (up to 6 months, inclusive) or long term (more than 6 months). If a study reported an outcome more than once in the same time point, only the last measure was considered.

### Searching for Studies

We conducted a broad search of the literature on January 28, 2025, using electronic and manual searches with no restriction of date, language, or status of publication.

We developed sensitive search strategies for the following electronic databases: ADOLEC (via Biblioteca Virtual em Saúde, BVS), Cochrane Central Register of Controlled Trials (CENTRAL, via Wiley), Epistemonikos, Excerpta Medica Database (Embase, via Elsevier), Latin American and Caribbean Health Sciences Literature (LILACS, via BVS), Medical Literature Analysis and Retrieval System Online (MEDLINE, via PubMed), and PsycINFO (via American Psychological Association, APA).

Additional searches for ongoing RCTs were conducted on ClinicalTrials.gov (www.clinicaltrials.gov) and The World Health Organization (WHO) International Clinical Trials Registry Platform (apps.who.int/trialsearch). The Data Archiving and Networked Services (DANS) was searched for grey literature. The manual search was performed by checking the reference lists of included studies and other relevant studies in the field. The search strategies for each database are presented in Table S1.

### Studies Selection and Data Extraction

The study selection process was conducted in two phases using the Rayyan platform.^23^ In the first phase, two reviewers independently assessed the titles and abstracts of all references retrieved by the search strategies. The references classified as “potentially eligible” were revised during the second phase, which consisted of assessing their full texts to confirm their eligibility. A third reviewer was consulted to solve inconsistencies, as necessary. The studies excluded during the second phase were listed, along with the reasons for each decision. The study selection process was detailed in a PRISMA flow diagram.

An independent pair of reviewers conducted data extraction using a pre‐established worksheet. Divergences in this process have been solved by consulting a third reviewer. We extracted the following data from each included study: general information from RCT (authors, publication year, funding sources, and conflict of interests), data from participants (sample size, mean age, ASD severity, treatment status considering whether they have had any previous treatment, and are currently undergoing treatment or have never undergone treatment), data from intervention/comparator (name, dose, route, scheme, and duration), and data from outcomes (name, measurement tool and time point, effect size estimates, confidence intervals, and variance measures).

### Risk of Bias Assessment

We assessed the risk of bias in the included studies using the Cochrane Risk of Bias tool (RoB tool), according to the recommendations of the Cochrane Handbook for Systematic Reviews of Interventions.^24^ The risk of bias was assessed by a pair of independent reviewers and disagreements would be solved by a third reviewer. Full justifications for each judgment were reported.

### Unit of Analysis

The unit of analysis was the individual participant of included studies.

### Heterogeneity Assessment

We considered the methodological and clinical diversity of included studies to judge heterogeneity. Statistical heterogeneity would be assessed using the Chi^2^ test (*P* < 0.1 would be used as a significant threshold) and I^2^ test (I^2^ > 50% would be used as an indicator of high inconsistency among RCTs). We planned additional analyses to explore reasons for heterogeneity and its impact on results obtained.

### Measures of Treatment Effect and Data Analysis

We presented risk ratios to assess dichotomous variables and mean differences for continuous variables, considering a 95% confidence interval in the analyses. We planned to perform a meta‐analysis in case of data availability and study homogeneity. In this case, as we expect some diversity among RCTs, we intend to apply a random effects model. For quantitative analysis, we would use the software STATA and Review Manager 5.4 software. Otherwise, narrative synthesis would be adopted.

### Additional Analysis

We planned the following additional analysis for confirming the robustness of results for primary outcomes:

- Subgroup analysis: (i) low versus high severity of ASD; (ii) pediatric versus adult participants; (iii) presence versus absence of other neurodevelopmental comorbidities.
- Sensitivity analysis: (i) fixed‐effect versus random‐effects model meta‐analysis. If the results of fixed‐effect meta‐analysis resulted in a different result, both would be reported, but the main conclusion would be based on random‐effects results; (ii) excluding from analysis those studies at high risk of bias on at least one domain of the RoB.

However, due to the paucity of studies, additional analyses were not conducted.

### Publication Bias Assessment

We planned to investigate publication bias by visual inspection of funnel plots, if at least 10 studies were included in a single meta‐analysis.

### Dealing with Missing Data

We contacted authors from included studies asking for missing data when relevant.

### Assessment of the Certainty of the Evidence

We assessed the certainty of the evidence using the Grading of Recommendations, Assessment, Development, and Evaluations (GRADE) approach^25^ that considers five domains: imprecision, inconsistency, indirectness, risk of bias, and publication bias. Based on these domains, each outcome was judged as very low, low, moderate, or high evidence certainty. The certainty of evidence was evaluated for each comparison and considering all outcomes. Reasons for each judgment were presented and the results were presented in summary of findings tables using GRADEpro GDT software.

## Results

### Results of the Search for Studies

The searches retrieved 1264 references. After the exclusion of 252 duplicates, we screened 1012 titles and abstracts. At this stage, 987 references were considered not eligible, and 25 were selected for full‐text analysis, after which 10 were excluded (Table S2). In total, this review included 15 reports from 11 studies, 4 being completed^26^, ^27^, ^28^, ^29^, ^30^ and 7 ongoing clinical trials.^31^, ^32^, ^33^, ^34^, ^35^, ^36^, ^37^ The flowchart of the selection process is presented in Figure S1.

### Characteristics of Included Studies

This systematic review included two parallel RCT^27^, ^29^ and the first phase of two cross‐over trials^26^, ^29^ which included 351 children and adolescents comparing different presentations of cannabis derivatives or their analogues with placebo for ASD. The five cannabis presentations studied were: whole plant extract, purified plant extract, extraction CBD (cannabidiol)/THC (tetrahydrocannabinol) (9:1 ratio), cannabidiol oil 100 mg/mL THC, and cannabidiol 100mg. The main characteristics of the completed and ongoing included studies are presented in Table 1 and Table S3, respectively.

**Table 1:** Main Characteristics of the Included Randomized Controlled Trials

Study and Location	Population (Sample Size)	Interventions	Comparators	Main Outcomes (Time Points)	Funding Source
Aran et al (2021)^26^ (NCT02956226) Israel	Children and adolescents (5 to 21 years) with ASD and moderate to serious disruptive behavior (n = 150)	Group 1: Whole plant extraction of cannabis dissolved in olive oil (167 mg/mL CBD and 8.35 mg/mL THC) (n = 50) Group 2: Purified cannabis extraction (99% pure CBD) dissolved in olive oil (167 mg/mL CBD and 8.35 mg/mL THC) (n = 50)	Olive oil: oral solution with a flavor and texture similar to the technology (n = 50)	Home Situations Questionnaire‐ASD CGI‐Improvement Social Responsiveness Scale‐2nd edition (SRS‐2) Autism Parenting Stress Index (APSI) Adverse events *Time point: 12 weeks (first phase)*	BOL Pharma, Revadim, Israel and the National Institute for Psychobiology in Israel (#203‐17‐18)
NCT04745026/2020‐002819‐21/GWND19189 United States, Australia, Canada, Germany, Spain, and United Kingdom	Children and adolescents (6 to 17 years) with weight at least 12 kg and ASD (n = 103)	Group 1: 5 mg/kg/day GWP42003‐P for 1 week and then 10 mg/kg/day GWP42003 oral solution (100 mg/mL CBD in sesame oil with anhydrous ethanol, ethanol 10% v/v, sweetener [sucralose]) (n = 49)	Placebo Matching oral solution containing sesame oil with anhydrous ethanol, sweetener (sucralose), strawberry flavoring, and beta carotene (n = 54)	Change from Baseline in ABC Subscale Scores Change from Baseline in VABS‐3 Scores CGI‐I Scores Change from Baseline in CGI‐S Scores *Time point: 12 weeks*	Jazz Pharmaceuticals
Parrella et al (2024) (ACTRN12622000437763) Australia	Children (5 to 12 years) with ASD (n = 34)	Group 1: Cannabidiol oil (30 mL bottle, total cannabinoid content per bottle 3000 mg, each milliliter of oil contained 100 mg of cannabidiol and THC not exceeding 1 mg/mL) (n = 17)	Placebo Formulated with medium‐chain triglyceride (MCT) excipients, matched in taste to the intervention (n = 17)	Social Responsiveness Scale – 2nd Edition (SRS‐2) Repetitive Behavior Scale‐Revised (RBS‐R) Vineland Adaptive Behavior Scale‐3 (VABS‐3) Pediatric Quality of Life Inventory (PedsQL) Behavior Rating Inventory of Executive Function ‐ Second Edition (BRIEF 2) Personal Wellbeing Index School Children (PWI‐3‐self‐report) Developmental Behavior Checklist‐2 (DBC‐2) Autism Parenting Stress Index (APSI) PROMIS EC Parent‐Report – Anxiety PROMIS EC Parent‐Report – Sleep Health PROMIS EC Parent‐Report – Social Relationships *Time point: 12 weeks (first phase)*	Medigrowth provided the product under investigation
Silva Júnior et al (2024) (RBR‐5wr2cqq) Brazil	Children (5 to 12 years) with ASD (mild, moderate or serious) (n = 64)	Group 1: Cannabis extraction with CBD 0.5% (5 mg/mL), with a THC and CBD proportion of 9:1 (n = 32)	Placebo Drops with the same consistency, color, odor, and other organoleptic characteristics as the intervention (n = 32)	Autism Treatment Evaluation Checklist (ATEC) Childhood Autism Rating Scale (CARS) Semi‐structured interview Safety and tolerability *Time point: 12 weeks*	None declared

ABC, Autistic Behavior Checklist; APSI, Autism Parenting Stress Index; ASD, autism spectrum disorder; ATEC, Autism Treatment Evaluation Checklist; BRIEF, Behavior Rating Inventory of Executive Function; CARS, Childhood Autism Rating Scale; CBD, cannabidiol; CGI, Clinical Global Impressions scale; DBC‐2, Developmental Behavior Checklist‐2; MCT, medium‐chain triglyceride; n, number of participants; PedsQL, Pediatric Quality of Life Inventory; PWI, Personal Wellbeing Index School Children; RBS‐R, Repetitive Behavior Scale‐Revised; SRS‐2, Social Responsiveness Scale‐2; THC, tetrahydrocannabinol; VABS, Vineland Adaptive Behavior Scale‐3.

### Risk of Bias of Included Studies

**Figure 1:**
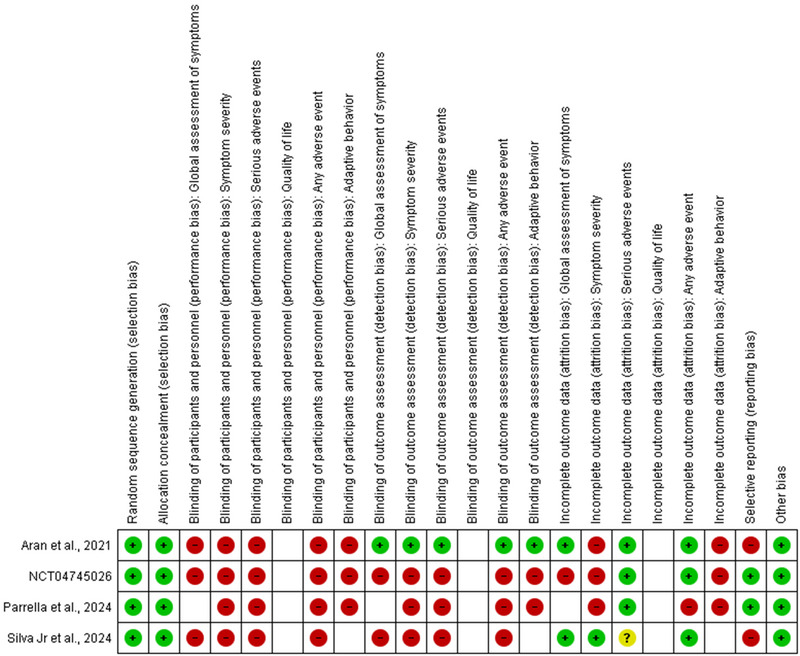
Summary of risk of bias assessment. Low risk of bias (green), some concerns (yellow), high risk (red). Missing judgments indicates the study did not assess the outcome.

Figure 1 presents the summary of risk of bias assessment of the four included studies using the Cochrane RoB tool.^24^

Overall, all studies are subject to a high risk of bias due to problems related mainly to the lack of blinding of participants and personnel. Although a placebo very similar to the intervention was used in the four studies, giving the caregiver the chance to increase the dosage according to their observation and judgment means that they have an important influence on the delivery of the intervention. Furthermore, since the parents/caregivers themselves reported the perceived improvements through semi‐structured interviews or other tools for most outcomes, the evaluation of these can be compromised by subjectivity. All risk of bias judgments and reasoning are presented in Table S4.

### Results of Included Studies

The results for each outcome are presented in Table 2 for each one of the included studies.

**Table 2:** Results From the Included Studies

Outcome	Aran et al (2021)^26^	NCT04745026	Parrella et al (2024)	Silva Junior et al (2024)
Global assessment of symptoms	Assessment tool: the proportion of participants that responded to be “much” or “very much” improved on the CGI‐I scale. Whole plant extract versus placebo: RR: 2.30 (95% CI 1.23 to 4.30); 1 RCT; 92 participants. Purified extract versus placebo: RR: 1.78 (95% CI 0.91 to 3.45); 1 RCT; 92 participants.	Assessment tool: the proportion of participants that responded to be “much” or “very much” improved on the CGI‐I scale. RR: 1.12 (95% CI 0.58 to 2.15); 1 RCT; 72 participants.	Outcome not assessed by the study.	Assessment tool: ATEC‐T. Score (0 to 179), lower values indicate less limitations. Data for this outcome could not be assessed due to inconsistencies between publications of the study. The authors were contacted, but no response was obtained.
Symptom severity	Assessment tool: SRS‐2 Score (0 to 195), lower values indicate less symptom severity.^a^ Whole plant extract versus placebo: MD: −11.0 (95% CI −24.47 to 2.47); 1 RCT; 65 participants. Purified extract versus placebo: MD: −15.00 (95% CI −27.46 to −2.54); 1 RCT; 69 participants.	Assessment tool: ABC Score (0 to 174), lower values indicate less symptom severity. Data for this outcome could not be assessed, as it was presented according to subscales. The total score was not presented. The authors were contacted, but no response was obtained.	Assessment tool: SRS‐2 Score (0 to 195), lower values indicate less symptom severity. MD: −14.00 (95% CI −39.94 to 11.94); 1 RCT; 27 participants.	Assessment tool: CARS Score (15 to 60), lower values indicate less symptom severity. Data for this outcome could not be assessed due to inconsistencies between publications of the study. The authors were contacted, but no response was obtained.
Serious adverse events	Assessment tool: proportion of participants with at least one serious adverse event.^a^ Whole plant extract versus placebo: RR: 1.09 (95% CI: 0.07 to 16.90); 1 RCT; 94 participants. Purified extract versus placebo: RR: 2.09 (95% CI: 0.20 to 22.24); 1 RCT; 96 participants.	Assessment tool: proportion of participants with at least one serious adverse event. RR: 2.20 (95% CI 0.21 to 23.56); 1 RCT; 103 participants.	The authors reported that they had not observed any serious adverse events.	The authors reported that they had not observed any serious adverse events.
Quality of life	Outcome not assessed by the study.	Outcome not assessed by the study.	Outcome not assessed by the study.	Outcome not assessed by the study.
Any adverse event	Assessment tool: proportion of participants with any adverse event.^a^ Whole plant extract versus placebo: RR: 1.06 (95% CI 0.93 to 1.21); 1 RCT; 96 participants. Purified extract versus placebo: RR: 1.09 (95% CI 0.97 to 1.23); 1 RCT; 98 participants.	Data for this outcome could not be assessed due to lack of clear data about the number/proportion of participants with any adverse event.	Assessment tool: number of participants with any adverse event. Group cannabis: 2/13 Group placebo: 0/14 1 RCT; 27 participants.	Assessment tool: number of participants with any adverse event: RR: 0.75 (95% CI 0.22 to 2.52); 1 RCT; 60 participants.
Adaptive behavior	Assessment tool: HSQ‐ASD Score (0 to 9), lower values indicating less symptom severity.^a^ Whole plant extract versus placebo: MD: −0.43 to (95% CI −1.20 to 0.34); 1 RCT; 79 participants. Purified extract versus placebo: MD: −0.40 to (95% CI −1.24 to 0.44); 1 RCT; 81 participants.	Assessment tool: VABS‐3 Score (20 to 140), lower values indicating less adaptive level. MD: ‐0.70 (95% CI −5.46 to 4.06); 1 RCT; 63 participants.	Assessment tool: VABS‐3 Score (20 to 140), lower values indicating less adaptive level. MD: 2.60 (95% CI −10.23 to 15.43); 1 RCT; 27 participants.	Outcome not assessed by the study.

ABC, Autistic Behavior Checklist; ATEC, Autism Treatment Evaluation Checklist; CARS, Childhood Autism Rating Scale; CGI, Clinical Global Impressions Scale; CI, confidence interval; HSQ‐ASD, Home Situations Questionnaire‐Autism Spectrum Disorder; MD, mean difference; RCT, randomized controlled trial; RR, relative risk; SRS‐2, Social Responsiveness Scale‐2; VABS‐3, Vineland Adaptive Behavior Scales‐3.

^a^ Results were calculated based on the raw data shared by the study authors.

From the four included RCTs, one had available outcome results after contact with authors.^26^ Inconsistencies were identified between different publications of the Silva Junior et al (2024)^29^ study for the outcomes “global assessment of symptoms” and “symptom severity.” The authors were contacted, but up to the time of writing this article there has been no response. Parrella et al (2024)^29^ and NCT04745026^27^ suffered mainly from loss to follow up: the first is a small study and any loss could have a major impact on its results, and the second lost different numbers of participants per outcome, even though they were assessed at the same time points.

The outcome “quality of life” was not assessed by any of the included studies and the study Parrella et al (2024)^29^ contributed with data for only three of the six planned outcomes for this review: symptom severity, any adverse event, and adaptive behavior.

As the studies evaluated five different formats of the intervention, it was not possible to pool the results into meta‐analyses.

### Certainty of the Evidence

We performed the assessment of the certainty of the evidence based on the GRADE approach. Overall, outcome domains across comparisons were judged to be at very low to low certainty of evidence. The reasons for downgrading were the high risk of bias of the included studies (especially the domains “blinding of participants and personnel,” “blinding of outcome assessors,” “incomplete outcome data,” and “selective reporting”) and imprecision related to some outcomes.

For the comparison of whole cannabis extract versus placebo, the certainty of evidence was considered very low to all but one outcome (global assessment of symptoms and low certainty of evidence). For the comparison of purified cannabis extract versus placebo, the certainty of evidence was considered very low for all outcomes. For the comparison of CBD oil (GWP42003‐P) versus placebo, the certainty of evidence was considered as very low for three outcomes (global assessment of symptoms, serious adverse events, and adaptive behavior) and for two the certainty could not be assessed due to lack of clear data for analysis (symptoms severity and any adverse event).

For the comparison Cannabis oil (Medigrowth CBD100) versus placebo, the certainty of evidence was considered as very low for three outcomes (symptom severity, any adverse event, and adaptive behavior), but the outcome serious adverse events were not assessed, as the study reported that no events occurred during its course. For the last comparison, cannabis extraction (CBD 0.5%) versus placebo, the certainty of evidence was considered as very low for the incidence of any adverse event. Global assessment of symptoms and symptom severity was not assessed due to inconsistencies between different publications of the study.

The certainty of evidence of each outcome for each comparison is presented in summary of findings tables, available in Table S5, with the respective reasons to downgrade each assessment of evidence obtained.

## Discussion

This systematic review identified 11 RCTs aiming to compare cannabis derivatives and their analogs with placebo for people with ASD, but only four had available outcome data for the present time. These RCTs intended to assess the efficacy and safety of different types of cannabis: whole plant extraction of cannabis dissolved in olive oil (167 mg/mL CBD [cannabidiol] and 8.35 mg/mL THC [tetrahydrocannabinol]),^26^ purified cannabis extraction (99% pure CBD) dissolved in olive oil (167 mg/mL CBD and 8.35 mg/mL THC),^26^ GWP42003 oral solution (100 mg/mL CBD in sesame oil with anhydrous ethanol, ethanol 10% v/v, sweetener),^27^ cannabidiol oil with the dosing of 10 mg/kg/day of cannabidiol (Medigrowth CBD100),^28^ and cannabis extraction with CBD 0.5% (5 mg/mL) with a THC and CBD proportion of 9:1.^29^

In total, the RCTs included 351 participants with different levels of severity of ASD. A very similar type of placebo was administered to all control groups, but as the caregivers were responsible for increasing the doses according to their observation and judgement of the participants’ needs, they had a very important influence on the implementation of the intervention, probably affecting the effect of the intervention. As all outcomes were subjective and assessed using self‐administered tools or semi‐structured interviews, the assessment of the outcomes may also have been affected.

The available results are from four studies assessing five different types of preparation and administration of cannabis (example given, one study calculated the dose according to the participant's weight and the other evaluated a fixed amount of drops). These studies also differ in terms of population characteristics (one study included children and adolescents with moderate to severe ASD according to the DSM‐V and the other included only children with ASD according to the attending physician at any degree of impairment), and outcomes (variety of tools and evaluations) which affected the possibility of this review to pool the obtained data.

Based on evidence of low‐certainty, whole cannabis extract may improve global assessment of symptoms, but the effects of whole cannabis extract in the incidence of symptom severity, serious adverse events, any adverse event, and adaptive behavior are uncertain. The effects of the purified cannabis on the global assessment of symptoms, symptom severity, serious adverse events, any adverse event, and adaptive behavior are uncertain. The effects of GWP42003 oral solution on the global assessment of symptoms, serious adverse events, and adaptive behavior are uncertain. The effects of the cannabis oil on the symptom severity, any adverse event, and adaptive behavior are uncertain. The effects of the cannabis extraction (CBD 0.5%) on the incidence of any adverse event is uncertain.

Uncertainties still remain for all cannabis preparations (safety and efficacy) due to high risk of bias, mainly related to lack of blinding of participants and personnel, blinding of outcome assessors and incomplete outcome data, and imprecision due to low number of participants and events. The certainty of evidence ranged from low to very low, suggesting that data from new studies may change these conclusions substantially. In addition, there is a need to carry out studies with the adult population: of the seven ongoing studies, only one is recruiting only participants over the age of 18.^34^

In a rapid search for RCTs assessing the effectiveness of cannabis derivatives and their analogues in MEDLINE via Pubmed, it was possible to notice that from the 19,400 references identified, 16,000 were published in the last 20 years. The amount of evidence from 2004 to 2024 increased more than five times. The increase in interest in the effects of cannabis assessed by RCTs also led to an increase in the production of systematic reviews in various kinds of conditions from neurological/psychological diagnoses (dementia, epilepsy, schizophrenia, and multiple sclerosis),^8^, ^9^, ^11^, ^38^ to pain (neuropathic pain, cancer pain, and fibromyalgia),^14^, ^39^, ^40^ and gastrointestinal diseases or symptoms (ulcerative colitis, Crohn's, nausea and vomiting related to chemotherapy).^13^, ^41^, ^42^

A common point between all these systematic reviews is that none has indicated that cannabis is effective or not with high certainty of evidence for any outcome analyzed and the same problems can be identified between the studies: imprecision, small sample size, and high risk of bias in at least one domain of the Cochrane RoB tool. Problems very similar to those identified in the RCTs included in this systematic review.

Another thing that these studies have in common is that they were carried out on people with diagnoses that may be considered difficult to manage and in which many other attempts of treatment had often been made, but without success. People diagnosed with ASD can fit into this type of patient. According to WHO, people with autism develop a complex range of symptoms and need a variety of medication, and psychological and social care integrated with their individual needs in order to lead to a more dignified life.^1^ However, there is also no evidence of high certainty about treatments for people with autism.

Systematic reviews evaluating different treatments for ASD, from pharmacological to behavioral interventions, for both the person with autism and their family members, identified similar problems that affected the certainty of the evidence obtained for the different outcomes evaluated: high risk of bias and imprecision.^43^, ^44^, ^45^, ^46^, ^47^, ^48^ This scenario makes decision making on the best treatment for ASD even more difficult, but at the same time opens space for evaluating different strategies, such as cannabis.

Considering the results of the studies included in this systematic review, one possible advantage of approving the administration of cannabis for ASD refers to the availability of a new treatment for refractory symptoms and moderate to severe cases of ASD. However, adverse events can be expected.

Regarding regulatory agencies, no evaluations of cannabis derivatives and their analogues specifically for ASD were found at the Canadian Agency for Drugs and Technologies in Health (CADTH), the UK's National Institute for Healthcare and Excellence (NICE), and the European Medicines Agency (EMA). In 2019, the FDA published a letter warning the industry about advertisements for products containing cannabidiol that are not approved for the treatment of toothache and earache in children, ASD, ADHD, and Parkinson's and Alzheimer's diseases.^49^

A previous systematic review evaluated cannabis or cannabinoids for ASD. Silva Junior et al (2022)^15^ included nine studies of varying designs with around 580 participants including children, adolescents, and adults. This review has limitations such as the inclusion of different types of studies to evaluate the effectiveness of an intervention, which did not allow for a quantitative analysis and introduced the possibility of various biases, however, states that it was possible to observe that cannabis improved symptoms such as anxiety, aggression, and irritability in people with ASD. In addition, problems were identified in locating studies (databases were missing, the search strategy was not reproducible, and there was no search of RCT registry databases), there was no assessment of risk of bias/quality of the included studies, and the certainty of evidence was not assessed.

The strengths of our systematic review were its methodological rigor, the sensitivity with which it searched for studies, the careful assessment of the included studies and the evidence obtained, and the transparency with which it was carried out. Its limitations are related to the fact that most of the included studies are ongoing, with data available from only four, and it is not viable to determine possible advantages, disadvantages, or differences between the different types of cannabis assessed and placebo. The protocol for the review originally planned to assess risk of bias of included RCTs using Cochrane RoB 2.0 tool, but due to methodological limitations of the tool, it was decided to use Cochrane Risk of Bias original version.^50^, ^51^, ^52^, ^53^ This change in planning was made prior to the studies' selection process.

Lastly, this systematic review has identified and brought together the main characteristics of completed and ongoing RCTs evaluating the effectiveness and safety of cannabis and its analogues for ASD. It was possible to present the current scenario and future perspectives on this new type of treatment that could impact the decision‐making process in clinical practice, especially for children and adolescents with difficult‐to‐manage symptoms and moderate to severe levels of ASD.

## Conclusions

The results found in this systematic review are from four completed RCTs assessing five different methods of preparation and administration of cannabis and its analogs for children and adolescents with ASD. The certainty of the evidence ranged from low to very low for all outcomes. In addition, seven ongoing studies included in this review do not yet have available data. The results presented here are still preliminary and should be used with caution in clinical practice. It is likely that the results from the ongoing studies will add robustness to the current findings.

In terms of implications for future research, the results of ongoing RCTs will most likely provide a new scenario for what is known so far about the benefits and risks of cannabis and its analogs for ASD. Questions about the best methods to prepare and administer cannabis and manage long‐term adverse events may also be answered.

For clinical practice, it is still difficult to talk about a clear recommendation for the use of cannabis for ASD outside of clinical trials. In addition, the costs associated with treatment still need to be evaluated.

## Author Contributions

**Rachel Riera**: Conceptualization; methodology; project administration; supervision; writing—review and editing. **Isabela Porto de Toledo**: Data curation; formal analysis; investigation; writing—original draft preparation. **Cecília Menezes Farinasso**: Data curation; investigation; writing—original draft preparation. **Rafael Leite Pacheco**: Conceptualization; project administration; validation; writing—original draft preparation. **Roberta Borges Silva**: Investigation; validation; writing—original draft preparation. **Verônica Colpani**: validation; writing—review and editing. **Ana Luiza Cabrera Martimbianco**: Validation; writing—review and editing. **Camila Monteiro Cruz**: Data curation; writing—original draft preparation. **Patrícia do Carmo Silva Parreira**: Data curation; writing—original draft preparation. **Carolina de Oliveira Cruz Latorraca**: conceptualization; data curation; formal analysis; methodology; visualization; writing—original draft preparation.

## Conflicts of Interest

The authors declare no conflicts of interest.

## Funding

This study was supported by Programa de Apoio ao Desenvolvimento Institucional do Sistema Único de Saúde (PROADI‐SUS) ‐ NUP (25000.175715/2023‐41), Brazilian Ministry of Health, Brazil. The supporter had no role in designing and conducting this study; or its collection, management, analysis, and interpretation of the data; preparation, review, or approval of the manuscript; and decision to submit the manuscript for publication.

## Supporting information



Supporting Information

Supporting Information

## Data Availability

All data analyzed in this review is available in the Supplemental Information.
