# Supplementary material for: Therapeutic Use of Cannabis Derivatives and Their Analogs for Autism Spectrum Disorder: A Systematic Review
Source: J Clin Pharmacol. 2025 Jul 2;65(11):1339–49. doi: 10.1002/jcph.70068 (PMC12555098; doi:10.1002/jcph.70068)
Supplement: Supplementary file 1 — Supporting Information [file JCPH-65-1339-s001.docx]

Therapeutic use of cannabis derivatives and their analogs for autism spectrum disorder: a systematic review.

Short title: Cannabis for autism: a systematic review.

# SUPPLEMENTARY FILES

Supplementary Figure S1. Flowchart of the selection process.


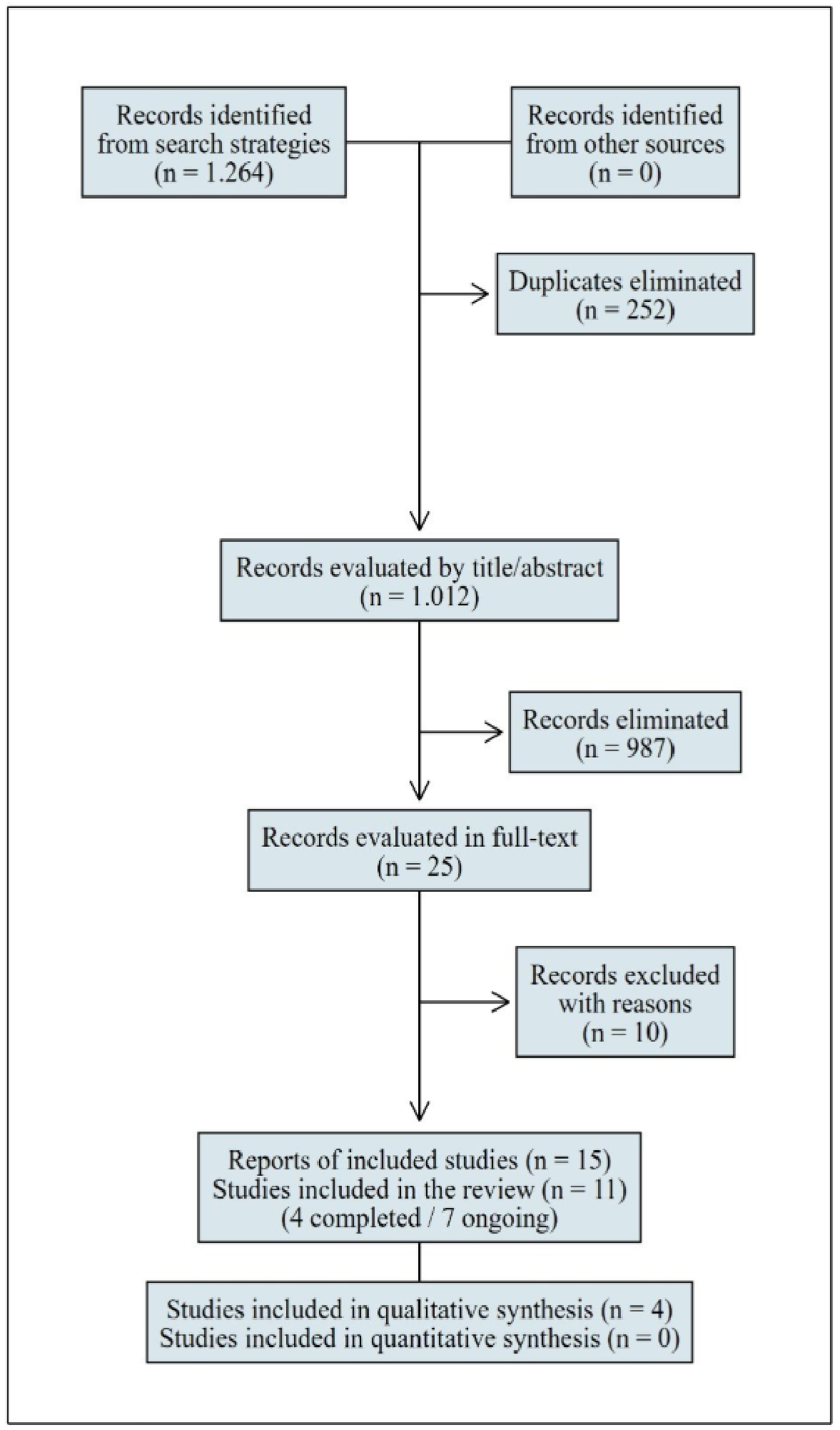


Supplementary Table S1. Search strategies for all eletronic databases considered (January 25^th^, 2025)

| **Database** | **Search Strategy** | **Results**  **(January 27^th^, 2025)** |
| --- | --- | --- |
| **MEDLINE via PubMed** | #1 "Autistic Disorder"[Mesh] OR (Autistic Disorder) OR (Disorder, Autistic) OR (Disorders, Autistic) OR (Kanner's Syndrome) OR (Kanner Syndrome) OR (Kanners Syndrome) OR (Autism, Infantile) OR (Infantile Autism) OR Autism OR (Autism, Early Infantile) OR (Early Infantile Autism) OR (Infantile Autism, Early)  #2 "Autism Spectrum Disorder"[Mesh] OR (Autism Spectrum Disorders) OR (Autistic Spectrum Disorder) OR (Autistic Spectrum Disorders) OR (Disorder, Autistic Spectrum) OR (Autistic Children) OR (Autistic People)  #3 "Asperger Syndrome"[Mesh] OR (Syndrome, Asperger) OR (Asperger's Disease) OR (Asperger's Diseases) OR (Aspergers Disease) OR (Disease, Asperger's) OR (Diseases, Asperger's) OR (Asperger Disease) OR (Asperger Diseases) OR (Disease, Asperger) OR (Diseases, Asperger) OR (Asperger Disorder) OR (Asperger Disorders) OR (Disorder, Asperger) OR (Disorders, Asperger) OR (Asperger's Disorder) OR (Aspergers Disorder) OR (Disorder, Asperger's) OR (Asperger's Syndrome) OR (Aspergers Syndrome) OR (Syndrome, Asperger's)  #4 "Child Development Disorders, Pervasive"[Mesh] OR (Child Development Disorders, Pervasive) OR (Pervasive Child Development Disorders) OR (Pervasive Development Disorders)  #5 #1 OR #2 OR #3 OR #4  #6 "Cannabinoids"[Mesh] OR Cannabinoid OR Cannabinoids  #7 "Cannabidiol"[Mesh] OR Cannabidiol OR "1,3-Benzenediol, 2-(3-methyl-6-(1-methylethenyl)-2-cyclohexen-1-yl)-5-pentyl-, (1R-trans)-" OR Epidiolex  #8 "Cannabinol"[Mesh] OR Cannabinol  #9 "Dronabinol"[Mesh] OR " delta(9)-THC" OR "9-ene-Tetrahydrocannabinol" OR "9 ene Tetrahydrocannabinol" OR THC OR "delta(1)-Tetrahydrocannabinol" OR "delta(1)-THC" OR "delta(9)-Tetrahydrocannabinol" OR Tetrahydrocannabinol OR "Tetrahydrocannabinol, (6a-trans)-Isomer" OR "Tetrahydrocannabinol, Trans-Isomer" OR "Tetrahydrocannabinol, Trans Isomer" OR "Tetrahydrocannabinol, (6aS-cis)-Isomer" OR "Tetrahydrocannabinol, Trans-(+-)-Isomer" OR Marinol OR Tetrahydrocannabidiol OR Mevatyl OR "delta-9-tetrahydrocannabinol"  #10 "Cannabis"[Mesh] OR Cannabis OR Cannabi OR (Hemp Plant) OR (Hemp Plants) OR Marihuana OR Marijuana OR (Cannabis indica) OR (Cannabis sativa) OR Hemp OR Hemps OR Hashish OR Hashishs OR Bhang OR Bhangs OR Ganja OR Ganjas  #11 "Cannabaceae"[Mesh] OR Cannabaceae  #12 "cannabidivarin" [Supplementary Concept] OR cannabidivarin  #13 "Medical Marijuana"[Mesh] OR (Medical Cannabis) OR (Medicinal Cannabis) OR (Marijuana Treatment) OR (Medicinal Marijuana) OR (Medical Cannabis) OR (Marijuana Dispensaries)  #14 "cannabis full spectrum" OR "cannabis full-spectrum" OR "full-spectrum cannabis" OR "full spectrum cannabis"  #15 "Full-Spectrum Hemp Extracts" OR "Full Spectrum Hemp Extracts" OR "Full-Spectrum Hemp Extract" OR "Full Spectrum Hemp Extract" OR "Full-Spectrum Hemp Oils" OR "Full Spectrum Hemp Oils" OR "Full-Spectrum Hemp Oil" OR "Full Spectrum Hemp Oil" OR "full-spectrum cannabis plant extract" OR "full spectrum cannabis plant extract" OR "full-spectrum cannabis plant extracts" OR "full-spectrum cannabis plant extracts"  #16 Sativex OR Nabilone OR Nabiximols OR Dexanabinol OR Marinol OR Namisol OR Syndros  #17 #6 OR #7 OR #8 OR #9 OR #10 OR #11 OR #12 OR #13 OR #14 OR #15 OR #16  #18 #5 AND #17 | 362 |
| **Cochrane Library**  **via Wiley** | #1 MeSH descriptor: [Autistic Disorder] explode all trees  #2 MeSH descriptor: [Autism Spectrum Disorder] explode all trees  #3 MeSH descriptor: [Asperger Syndrome] explode all trees  #4 MeSH descriptor: [Child Development Disorders, Pervasive] explode all trees  #5 (Autistic Disorder) OR (Autism Spectrum Disorder) OR (Asperger Syndrome) OR (Disorder, Autistic) OR (Disorders, Autistic) OR (Kanner's Syndrome) OR (Kanner Syndrome) OR (Kanners Syndrome) OR (Autism, Infantile) OR (Infantile Autism) OR Autism OR (Autism, Early Infantile) OR (Early Infantile Autism) OR (Infantile Autism, Early) OR (Autism Spectrum Disorders) OR (Autistic Spectrum Disorder) OR (Autistic Spectrum Disorders) OR (Disorder, Autistic Spectrum) OR (Syndrome, Asperger) OR (Asperger's Disease) OR (Asperger's Diseases) OR (Aspergers Disease) OR (Disease, Asperger's) OR (Diseases, Asperger's) OR (Asperger Disease) OR (Asperger Diseases) OR (Disease, Asperger) OR (Diseases, Asperger) OR (Asperger Disorder) OR (Asperger Disorders) OR (Disorder, Asperger) OR (Disorders, Asperger) OR (Asperger's Disorder) OR (Aspergers Disorder) OR (Disorder, Asperger's) OR (Asperger's Syndrome) OR (Aspergers Syndrome) OR (Syndrome, Asperger's) OR (Child Development Disorders, Pervasive) OR (Pervasive Child Development Disorders) OR (Pervasive Development Disorders)  #6 #1 OR #2 OR #3 OR #4 OR #5  #7 MeSH descriptor: [Cannabinoids] explode all trees  #8 MeSH descriptor: [Cannabidiol] explode all trees  #9 MeSH descriptor: [Cannabinol] explode all trees  #10 MeSH descriptor: [Dronabinol] explode all trees  #11 MeSH descriptor: [Cannabis] explode all trees  #12 MeSH descriptor: [Cannabaceae] explode all trees  #13 MeSH descriptor: [Medical Marijuana] explode all trees  #14 Cannabinoid OR Cannabinoids OR Cannabidiol OR "1,3-Benzenediol, 2-(3-methyl-6-(1-methylethenyl)-2-cyclohexen-1-yl)-5-pentyl-, (1R-trans)-" OR Epidiolex OR Cannabinol OR "delta(9)-THC" OR "9-ene-Tetrahydrocannabinol" OR "9 ene Tetrahydrocannabinol" OR THC OR "delta(1)-Tetrahydrocannabinol" OR "delta(1)-THC" OR "delta(9)-Tetrahydrocannabinol" OR Tetrahydrocannabinol OR "Tetrahydrocannabinol, (6a-trans)-Isomer" OR "Tetrahydrocannabinol, Trans-Isomer" OR "Tetrahydrocannabinol, Trans Isomer" OR "Tetrahydrocannabinol, (6aS-cis)-Isomer" OR "Tetrahydrocannabinol, Trans-(+-)-Isomer" OR Marinol OR Tetrahydrocannabidiol OR Mevatyl OR "delta-9-tetrahydrocannabinol" OR Cannabis OR Cannabi OR "Hemp Plant" OR "Hemp Plants" OR Marihuana OR Marijuana OR "Cannabis indica" OR "Cannabis sativa" OR Hemp OR Hemps OR Hashish OR Hashishs OR Bhang OR Bhangs OR Ganja OR Ganjas OR Cannabaceae OR cannabidivarin OR (Medical Cannabis) OR (Medicinal Cannabis) OR (Marijuana Treatment) OR (Medicinal Marijuana) OR (Medical Cannabis) OR (Marijuana Dispensaries) OR (cannabis full-spectrum) OR (full-spectrum cannabis) OR (full spectrum cannabis) OR (Full-Spectrum Hemp Extracts) OR (Full Spectrum Hemp Extracts) OR (Full-Spectrum Hemp Extract) OR (Full Spectrum Hemp Extract) OR (Full-Spectrum Hemp Oils) OR (Full Spectrum Hemp Oils) OR (Full-Spectrum Hemp Oil) OR (Full Spectrum Hemp Oil) OR (full-spectrum cannabis plant extract) OR (full spectrum cannabis plant extract) OR (full-spectrum cannabis plant extracts) OR (full-spectrum cannabis plant extracts) OR Sativex OR Nabilone OR Nabiximols OR Dexanabinol OR Marinol OR Namisol OR Syndros  #15 #7 OR #8 OR #9 OR #10 OR #11 OR #12 OR #13 OR #14  #16 #6 AND #15 | 44 |
| **EMBASE via Elsevier** | #1 'asperger syndrome'/exp OR 'asperger disorder' OR 'asperger`s disorder' OR 'asperger`s syndrome' OR 'aspergers disorder' OR 'aspergers syndrome' OR 'high functioning autism' OR 'asperger syndrome'  #2 'autism'/exp OR 'autism spectrum disorder' OR 'autism, early infantile' OR 'autism, infantile' OR 'autistic child' OR 'autistic children' OR 'autistic disorder' OR 'autistic spectrum disorder' OR 'child development disorders, pervasive' OR 'childhood autism' OR 'classical autism' OR 'early infantile autism' OR 'infantile autism' OR 'infantile autism, early' OR 'kanner syndrome' OR 'pdd (pervasive developmental disorder)' OR 'pervasive child development disorders' OR 'pervasive developmental disorder' OR 'pervasive developmental disorders' OR 'typical autism' OR 'autism' OR 'autistic people'  #3 #1 OR #2  #4 'cannabinoid'/exp OR 'cannabinoids' OR 'cannabinoid'  #5 'cannabidiol'/exp OR '2 (6 isopropenyl 3 methylcyclohex 2 en 1 yl) 5 pentylbenzene 1, 3 diol' OR '2 (6 isopropenyl 3 methylcyclohex 2 enyl) 5 pentylbenzene 1, 3 diol' OR '2 [3 methyl 6 (1 methylethenyl) 2 cyclohexen 1 yl] 5 pentyl 1, 3 benzenediol' OR '2 [3 methyl 6 (prop 1 en 2 yl) cyclohex 2 en 1 yl] 5 pentylbenzene 1, 3 diol' OR '2 para mentha 1, 8 dien 3 yl 5 pentylresorcinol' OR '5` methyl 4 pentyl 2` (prop 1 en 2 yl) 1`, 2`, 3`, 4` tetrahydrobiphenyl 2, 6 diol' OR 'a 1002 n5s' OR 'a1002n5s' OR 'btx 1204' OR 'btx 1308' OR 'btx 1503' OR 'btx 1702' OR 'btx 1801' OR 'btx1204' OR 'btx1308' OR 'btx1503' OR 'btx1702' OR 'btx1801' OR 'cardiolrx' OR 'epidiolex' OR 'epidyolex' OR 'gwp 42003' OR 'gwp 42003p' OR 'gwp42003' OR 'gwp42003p' OR 'nabidiolex' OR 'nantheia' OR 'oravexx' OR 'rad 011' OR 'rad011' OR 'trans cannabidiol' OR 'zygel' OR 'zyn 002' OR 'zyn002' OR 'cannabidiol'  #6 'cannabinol'/exp OR '3 pentyl 6, 6, 9 trimethyl (6h) dibenzopyran 1 ol' OR 'cannabinol'  #7 'dronabinol'/exp OR '1 trans delta 9 tetrahydrocannabinol' OR '3 pentyl 6, 6, 9 trimethyl 6a, 7, 8, 10a tetrahydro 6h dibenzo [b, d] pyran 1 ol' OR '6, 6, 9 trimethyl 3 pentyl 6a, 7, 8, 10a tetrahydrobenzo [c] chromen 1 ol' OR 'adversa' OR 'bx 1' OR 'bx1' OR 'delta 1 3, 4 trans tetrahydrocannabinol' OR 'delta 1 tetrahydrocannabinol' OR 'delta 1 trans tetrahydrocannabinol' OR 'delta 1, 2 tetrahydrocannabinol' OR 'delta 9 tetrahydrocannabinol' OR 'delta 9 trans tetrahydrocannabinol' OR 'delta1 3, 4 trans tetrahydrocannabinol' OR 'delta1 cis tetrahydrocannabinol' OR 'delta1 tetrahydrocannabinol' OR 'delta1 thc' OR 'delta1 trans tetrahydrocannabinol' OR 'delta9 tetrahydro cannabinol' OR 'delta9 tetrahydrocannabinol' OR 'delta9 trans tetrahydrocannabinol' OR 'ea 1477' OR 'ea1477' OR 'l delta 9 trans tetrahydrocannabinol' OR 'levo delta 1 tetrahydrocannabinol' OR 'levo delta 9 tetrahydrocannabinol' OR 'marinol' OR 'ppp 002' OR 'ppp002' OR 'qcd 84924' OR 'reduvo' OR 'relivar' OR 'syndros' OR 'tetrahydrocannabinol 1 ene' OR 'tetrahydrocannabinol delta1' OR 'tetrahydrocannabinol delta9' OR 'tetranabinex' OR 'trans delta 9 tetrahydrocannabinol' OR 'u1 tetrahydrocannabinol' OR 'u9 tetrahydrocannabinol' OR 'dronabinol'  #8 'cannabis'/exp OR 'bhang' OR 'cannabis alkaloid' OR 'cannabis constituent' OR 'cannabis extract' OR 'cannabis herba' OR 'cannabis leaf' OR 'cannabis sativa extract' OR 'cannabis sativa leaf' OR 'cannabis sativa resin' OR 'cannador' OR 'charas' OR 'ganja' OR 'ganjah' OR 'hashish' OR 'hashish oil' OR 'hemp extract' OR 'herba cannabis' OR 'indian bhang' OR 'indian ganja' OR 'marihuana' OR 'marijuana' OR 'mexican marihuana' OR 'cannabis'  #9 'cannabaceae'/exp OR 'cannabaceae'  #10 'cannabidivarin'/exp OR '2 (3 methyl 6 (prop 1 en 2 yl) cyclohex 2 enyl) 5 propylbenzene 1, 3 diol' OR '2 (6 isopropenyl 3 methyl 2 cyclohexen 1 yl) 5 propyl 1, 3 benzenediol' OR '2 [3 methyl 6 (1 methylethenyl) 2 cyclohexen 1 yl] 5 propyl 1, 3 benzenediol' OR 'gwp 42006' OR 'gwp42006' OR 'cannabidivarin'  #11 'medical cannabis'/exp OR 'medical marihuana' OR 'medical marijuana' OR 'medicinal cannabis' OR 'medicinal marihuana' OR 'medicinal marijuana' OR 'medical cannabis'  #12 'cannabis full spectrum' OR 'cannabis full-spectrum' OR 'full-spectrum cannabis' OR 'full spectrum cannabis' OR 'full-spectrum hemp extracts' OR 'full spectrum hemp extracts' OR 'full-spectrum hemp extract' OR 'full spectrum hemp extract' OR 'full-spectrum hemp oils' OR 'full spectrum hemp oils' OR 'full-spectrum hemp oil' OR 'full spectrum hemp oil' OR 'full-spectrum cannabis plant extract' OR 'full spectrum cannabis plant extract' OR 'full-spectrum cannabis plant extracts'  #13 sativex OR nabilone OR nabiximols OR dexanabinol OR marinol OR namisol OR syndros  #14 #4 OR #5 OR #6 OR #7 OR #8 OR #9 OR #10 OR #11 OR #12 OR #13  #15 #3 AND #14  #16 #15 AND [embase]/lim NOT ([embase]/lim AND [medline]/lim) | 437 |
| **LILACS via BVS** | #1 MH:"Transtorno Autístico" OR MH:"Trastorno Autístico" OR MH:"Autistic Disorder" OR (Transtorno Autístico) OR (Trastorno Autístico) OR (Autistic Disorder) OR (Síndrome de Kanner) OR (Autismo Infantil) OR Autismo OR (Early Infantile Autism) OR Autism OR (Infantile Autism) OR (Kanners Syndrome) OR (Kanner Syndrome) OR (Kanner's Syndrome) OR MH:F03.625.164.113.500$ OR MH:"Transtorno do Espectro Autista" OR MH:"Trastorno del Espectro Autista" OR MH:"Autism Spectrum Disorder" OR (Transtorno do Espectro Autista) OR (Trastorno del Espectro Autista) OR (Autism Spectrum Disorder) OR (Transtorno de Espectro Autista) OR (Transtorno do Espectro do Autismo) OR (Autism Spectrum Disorders) OR (Autistic Spectrum Disorder) OR (Autistic Spectrum Disorders) OR MH:F03.625.164.113$ OR MH:"Síndrome de Asperger" OR MH:"Asperger Syndrome" OR (Síndrome de Asperger) OR (Asperger Syndrome) OR (Asperger Disorders) OR (Aspergers Syndrome) OR (Asperger's Syndrome) OR (Aspergers Disorder) OR (Asperger's Disorder) OR (Asperger Disorder) OR (Asperger Diseases) OR (Asperger Disease) OR (Aspergers Disease) OR (Asperger's Diseases) OR (Asperger's Disease) OR MH:F03.625.164.113.250$ OR MH:"Transtornos Globais do Desenvolvimento Infantil" OR MH:"Trastornos Generalizados del Desarrollo Infantil" OR MH:"Child Development Disorders, Pervasive" OR (Transtornos Globais do Desenvolvimento Infantil) OR (Trastornos Generalizados del Desarrollo Infantil) OR (Transtornos Globais do Desenvolvimento da Criança) OR (Transtornos Globais do Desenvolvimento) OR (Pervasive Child Development Disorders) OR (Pervasive Development Disorders) OR (Trastornos Generalizados del Desarrollo del Niño) OR (Trastornos Generalizados del Desarrollo) OR MH:F03.625.164$  AND  #2 MH:Canabinoides OR MH:Cannabinoides OR MH:Cannabinoids OR Canabinoides OR Cannabinoides OR Cannabinoids OR Canabinoide OR Cannabinoid OR Cannabinoide OR MH:D02.455.849.090$ OR MH:Canabidiol OR MH:Cannabidiol OR Canabidiol OR Cannabidiol OR "1,3-Benzenodiol, 2- (3-metil-6- (1-metiletenil) -2-cicloexen-1-Il) -5-pentil-, (1R-trans) -" OR Epidiolex OR MH:D02.455.849.090.100$ OR MH:Cannabinol OR MH:Canabinol OR Cannabinol OR Canabinol OR MH:D02.455.849.090.110$ OR MH:Dronabinol OR Dronabinol OR "delta (9)-THC" OR Tetraidrocanabinol OR "Tetra-Hidrocanabinol" OR THC OR "9-ene-Tetrahydrocannabinol" OR "9 ene Tetrahydrocannabinol" OR "delta(1)-Tetrahydrocannabinol" OR "delta(1)-THC" OR "delta(9)-Tetrahydrocannabinol" OR Tetrahydrocannabinol OR "Tetrahydrocannabinol, (6a-trans)-Isomer" OR "Tetrahydrocannabinol, Trans-Isomer" OR "Tetrahydrocannabinol, Trans Isomer" OR "Tetrahydrocannabinol, (6aS-cis)-Isomer" OR "Tetrahydrocannabinol, Trans-(+-)-Isomer" OR Marinol OR "Tetrahydrocannabinol, (6aR-cis)-Isomer" OR Tetrahidrocannabinol OR Tetrahidrocanabinol OR MH:D02.455.849.090.810$ OR MH:Cannabis OR Cannabis OR Cannabi OR Cânabe OR Cânabis OR Cânave OR "Linho-Cânhamo" OR Maconha OR (Cannabis indica) OR (Cannabis sativa indica) OR "Cânhamo-da-Índia" OR Ganja OR Haxixe OR Cânhamo OR Bangue OR (Cannabis sativa) OR (Cannabis chinensis) OR (Hemp Plant) OR (Hemp Plants) OR Marihuana OR Marijuana OR Ganjas OR Hashish OR Hashishs OR Hemp OR Hemps OR Bhang OR Bhangs OR Mariguana OR Ganga OR Hachís OR Cáñamo OR MH:B01.875.800.575.912.250.859.937.055.500$ OR MH:HP4.018.189.205$ OR MH:Cannabaceae OR Cannabaceae OR Canabidáceas OR Canabáceas OR MH:B01.875.800.575.912.250.859.937.055$ OR MH:"Maconha Medicinal" OR MH:"Marihuana Medicinal" OR MH:"Medical Marijuana" OR (Maconha Medicinal) OR (Marihuana Medicinal) OR (Medical Marijuana) OR (Medical Cannabis) OR (Medicinal Marijuana) OR (Marijuana Treatment) OR (Medicinal Cannabis) OR (Marijuana Dispensaries) OR MH:D26.528$ OR cannabidivarin OR (cannabis full spectrum) OR (cannabis full-spectrum) OR (full-spectrum cannabis) OR (full spectrum cannabis) OR (Full-Spectrum Hemp Extracts) OR (Full Spectrum Hemp Extracts) OR (Full-Spectrum Hemp Extract) OR (Full Spectrum Hemp Extract) OR (Full-Spectrum Hemp Oils) OR (Full Spectrum Hemp Oils) OR (Full-Spectrum Hemp Oil) OR (Full Spectrum Hemp Oil) OR (full-spectrum cannabis plant extract) OR (full spectrum cannabis plant extract) OR (full-spectrum cannabis plant extracts) OR (full-spectrum cannabis plant extracts) OR Sativex OR Nabilone OR Nabiximols OR Dexanabinol OR Marinol OR Namisol OR Syndros OR Mevatyl  *In* LILACS, BRISA, BDENF, IBECS, Coleciona SUS | 27 |
| **PsycINFO via APA** | ((IndexTermsFilt: ("Autism Spectrum Disorders")) *OR* (Any Field: (Autistic Disorder)) *OR* (Any Field: (Autism Spectrum Disorder)) *OR* (Any Field: (Asperger Syndrome)) *OR* (Any Field: (Disorder, Autistic)) *OR* (Any Field: (Disorders, Autistic)) *OR* (Any Field: (Kanner's Syndrome)) *OR* (Any Field: (Kanner Syndrome)) *OR* (Any Field: (Kanners Syndrome)) *OR* (Any Field: (Autism, Infantile)) *OR* (Any Field: (Infantile Autism)) *OR* Any Field: (Autism) *OR* (Any Field: (Autism, Early Infantile)) *OR* (Any Field: (Early Infantile Autism)) *OR* (Any Field: (Infantile Autism, Early)) *OR* (Any Field: (Autism Spectrum Disorders)) *OR* (Any Field: (Autistic Spectrum Disorder)) *OR* (Any Field: (Autistic Spectrum Disorders)) *OR* (Any Field: (Disorder, Autistic Spectrum)) *OR* (Any Field: (Syndrome, Asperger)) *OR* (Any Field: (Asperger's Disease)) *OR* (Any Field: (Asperger's Diseases)) *OR* (Any Field: (Aspergers Disease)) *OR* (Any Field: (Disease, Asperger's)) *OR* (Any Field: (Diseases, Asperger's)) *OR* (Any Field: (Asperger Disease)) *OR* (Any Field: (Asperger Diseases)) *OR* (Any Field: (Disease, Asperger)) *OR* (Any Field: (Diseases, Asperger)) *OR* (Any Field: (Asperger Disorder)) *OR* (Any Field: (Asperger Disorders)) *OR* (Any Field: (Disorder, Asperger)) *OR* (Any Field: (Disorders, Asperger)) *OR* (Any Field: (Asperger's Disorder)) *OR* (Any Field: (Aspergers Disorder)) *OR* (Any Field: (Disorder, Asperger's)) *OR* (Any Field: (Asperger's Syndrome)) *OR* (Any Field: (Aspergers Syndrome)) *OR* (Any Field: (Syndrome, Asperger's)) *OR* (Any Field: (Child Development Disorders, Pervasive)) *OR* (Any Field: (Pervasive Child Development Disorders)) *OR* (Any Field: (Pervasive Development Disorders))) *AND* ((IndexTermsFilt: ("Cannabinoids") *OR* IndexTermsFilt: ("Endocannabinoids") *OR* IndexTermsFilt: ("Cannabis") *OR* IndexTermsFilt: ("Cannabidiol") *OR* IndexTermsFilt: ("Tetrahydrocannabinol") *OR* IndexTermsFilt: ("Medical Marijuana")) *OR* (Any Field: (Cannabinoid) *OR* Any Field: (Cannabinoids) *OR* Any Field: (Cannabidiol) *OR* Any Field: ("1,3-Benzenediol, 2-(3-methyl-6-(1-methylethenyl)-2-cyclohexen-1-yl)-5-pentyl-, (1R-trans)-") *OR* Any Field: (Epidiolex) *OR* Any Field: (Cannabinol) *OR* Any Field: ("delta(9)-THC") *OR* Any Field: ("9-ene-Tetrahydrocannabinol") *OR* Any Field: ("9 ene Tetrahydrocannabinol") *OR* Any Field: (THC) *OR* Any Field: ("delta(1)-Tetrahydrocannabinol") *OR* Any Field: ("delta(1)-THC") *OR* Any Field: ("delta(9)-Tetrahydrocannabinol") *OR* Any Field: (Tetrahydrocannabinol) *OR* Any Field: ("Tetrahydrocannabinol, (6a-trans)-Isomer") *OR* Any Field: ("Tetrahydrocannabinol, Trans-Isomer") *OR* Any Field: ("Tetrahydrocannabinol, Trans Isomer") *OR* Any Field: ("Tetrahydrocannabinol, (6aS-cis)-Isomer") *OR* Any Field: ("Tetrahydrocannabinol, Trans-(+-)-Isomer") *OR* Any Field: (Marinol) *OR* Any Field: (Tetrahydrocannabidiol) *OR* Any Field: (Mevatyl) *OR* Any Field: ("delta-9-tetrahydrocannabinol") *OR* Any Field: (Cannabis) *OR* Any Field: (Cannabi) *OR* Any Field: ("Hemp Plant") *OR* Any Field: ("Hemp Plants") *OR* Any Field: (Marihuana) *OR* Any Field: (Marijuana) *OR* Any Field: ("Cannabis indica") *OR* Any Field: ("Cannabis sativa") *OR* Any Field: (Hemp) *OR* Any Field: (Hemps) *OR* Any Field: (Hashish) *OR* Any Field: (Hashishs) *OR* Any Field: (Bhang) *OR* Any Field: (Bhangs) *OR* Any Field: (Ganja) *OR* Any Field: (Ganjas) *OR* Any Field: (Cannabaceae) *OR* Any Field: (cannabidivarin) *OR* (Any Field: (Medical Cannabis)) *OR* (Any Field: (Medicinal Cannabis)) *OR* (Any Field: (Marijuana Treatment)) *OR* (Any Field: (Medicinal Marijuana)) *OR* (Any Field: (Medical Cannabis)) *OR* (Any Field: (Marijuana Dispensaries)) *OR* (Any Field: (cannabis full-spectrum)) *OR* (Any Field: (full-spectrum cannabis)) *OR* (Any Field: (full spectrum cannabis)) *OR* (Any Field: (Full-Spectrum Hemp Extracts)) *OR* (Any Field: (Full Spectrum Hemp Extracts)) *OR* (Any Field: (Full-Spectrum Hemp Extract)) *OR* (Any Field: (Full Spectrum Hemp Extract)) *OR* (Any Field: (Full-Spectrum Hemp Oils)) *OR* (Any Field: (Full Spectrum Hemp Oils)) *OR* (Any Field: (Full-Spectrum Hemp Oil)) *OR* (Any Field: (Full Spectrum Hemp Oil)) *OR* (Any Field: (full-spectrum cannabis plant extract)) *OR* (Any Field: (full spectrum cannabis plant extract)) *OR* (Any Field: (full-spectrum cannabis plant extracts)) *OR* (Any Field: (full-spectrum cannabis plant extracts)) *OR* Any Field: (Sativex) *OR* Any Field: (Nabilone) *OR* Any Field: (Nabiximols) *OR* Any Field: (Dexanabinol) *OR* Any Field: (Marinol) *OR* Any Field: (Namisol) *OR* Any Field: (Syndros))) | 243 |
| **ADOLEC via BVS** | Autismo OR Autism | 3 |
| **Epistemonikos** | (Cannabinoid OR Cannabinoids OR Cannabidiol OR "1,3-Benzenediol, 2-(3-methyl-6-(1-methylethenyl)-2-cyclohexen-1-yl)-5-pentyl-, (1R-trans)-" OR Epidiolex OR Cannabinol OR "delta(9)-THC" OR "9-ene-Tetrahydrocannabinol" OR "9 ene Tetrahydrocannabinol" OR THC OR "delta(1)-Tetrahydrocannabinol" OR "delta(1)-THC" OR "delta(9)-Tetrahydrocannabinol" OR Tetrahydrocannabinol OR "Tetrahydrocannabinol, (6a-trans)-Isomer" OR "Tetrahydrocannabinol, Trans-Isomer" OR "Tetrahydrocannabinol, Trans Isomer" OR "Tetrahydrocannabinol, (6aS-cis)-Isomer" OR "Tetrahydrocannabinol, Trans-(+-)-Isomer" OR Marinol OR Tetrahydrocannabidiol OR Mevatyl OR "delta-9-tetrahydrocannabinol" OR Cannabis OR Cannabi OR "Hemp Plant" OR "Hemp Plants" OR Marihuana OR Marijuana OR "Cannabis indica" OR "Cannabis sativa" OR Hemp OR Hemps OR Hashish OR Hashishs OR Bhang OR Bhangs OR Ganja OR Ganjas OR Cannabaceae OR cannabidivarin OR (Medical Cannabis) OR (Medicinal Cannabis) OR (Marijuana Treatment) OR (Medicinal Marijuana) OR (Medical Cannabis) OR (Marijuana Dispensaries) OR (cannabis full-spectrum) OR (full-spectrum cannabis) OR (full spectrum cannabis) OR (Full-Spectrum Hemp Extracts) OR (Full Spectrum Hemp Extracts) OR (Full-Spectrum Hemp Extract) OR (Full Spectrum Hemp Extract) OR (Full-Spectrum Hemp Oils) OR (Full Spectrum Hemp Oils) OR (Full-Spectrum Hemp Oil) OR (Full Spectrum Hemp Oil) OR (full-spectrum cannabis plant extract) OR (full spectrum cannabis plant extract) OR (full-spectrum cannabis plant extracts) OR (full-spectrum cannabis plant extracts) OR Sativex OR Nabilone OR Nabiximols OR Dexanabinol OR Marinol OR Namisol OR Syndros) AND ((Autistic Disorder) OR (Autism Spectrum Disorder) OR (Asperger Syndrome) OR (Disorder, Autistic) OR (Disorders, Autistic) OR (Kanner's Syndrome) OR (Kanner Syndrome) OR (Kanners Syndrome) OR (Autism, Infantile) OR (Infantile Autism) OR Autism OR (Autism, Early Infantile) OR (Early Infantile Autism) OR (Infantile Autism, Early) OR (Autism Spectrum Disorders) OR (Autistic Spectrum Disorder) OR (Autistic Spectrum Disorders) OR (Disorder, Autistic Spectrum) OR (Syndrome, Asperger) OR (Asperger's Disease) OR (Asperger's Diseases) OR (Aspergers Disease) OR (Disease, Asperger's) OR (Diseases, Asperger's) OR (Asperger Disease) OR (Asperger Diseases) OR (Disease, Asperger) OR (Diseases, Asperger) OR (Asperger Disorder) OR (Asperger Disorders) OR (Disorder, Asperger) OR (Disorders, Asperger) OR (Asperger's Disorder) OR (Aspergers Disorder) OR (Disorder, Asperger's) OR (Asperger's Syndrome) OR (Aspergers Syndrome) OR (Syndrome, Asperger's) OR (Child Development Disorders, Pervasive) OR (Pervasive Child Development Disorders) OR (Pervasive Development Disorders)) | 108 |
| **DANS** | ("Autistic Disorder" OR "Autism Spectrum Disorder" OR "Asperger Syndrome" OR "Kanner's Syndrome" OR "Kanner Syndrome" OR "Kanners Syndrome" OR "Infantile Autism" OR Autism OR "Asperger's Disease") AND (Cannabidiol OR Cannabinoid OR Dronabinol OR Cannabis OR Cannabinoids OR Cannabaceae OR Cannabidivarin) | 6 |
| **Clinicaltrials.gov** | Condition or disease: (Autistic Disorder) OR (Autism Spectrum Disorder) OR (Asperger Syndrome) OR (Kanner Syndrome) OR (Infantile Autism) OR Autism OR (Early Infantile Autism) OR Autistic OR (Pervasive Child Development Disorders)  Intervention/treatment: Cannabidiol OR Cannabinol OR Cannabinoid OR Dronabinol OR Cannabis OR Cannabaceae OR Epidiolex OR Tetrahydrocannabinol OR Marinol OR Marijuana OR "Medicinal Cannabis" OR Cannabidivarin | 18 |
| **WHO-ICRTP** | Condition: (Autistic Disorder) OR (Autism Spectrum Disorder) OR (Asperger Syndrome) OR (Kanner Syndrome) OR (Infantile Autism) OR Autism OR (Early Infantile Autism) OR Autistic OR (Pervasive Child Development Disorders)  Intervention: Cannabidiol OR Cannabinol OR Cannabinoid OR Dronabinol OR Cannabis OR Cannabinoids OR Cannabaceae OR Epidiolex OR Tetrahydrocannabinol OR Marinol OR Marijuana OR Marihuana OR Hemp OR Hashish OR Bhang OR Ganja OR "Medicinal Cannabis" OR "Medical marijuana" OR Cannabidivarin OR Sativex OR Nabilone OR Nabiximols OR Dexanabinol OR Namisol OR Syndros OR Mevatyl  Recruitment status: ALL | 16 |
| **Total** | | 1.264 |

Supplementary Table S2. Excluded studies with reasons for exclusion

| **Wrong study design** |
| --- |
| 1. Aung-Din, R. Cannabinoid therapy neurodirect effectstm cbd: non-systemic cannabidiol for autism spectrum disorder. 2020. Disponível em: <https://drug-dev.com/cannabinoid-therapy-neurodirect-effectstm-cbd-non-systemic-cannabidiol-for-autism-spectrum-disorder/> 2. Castellanos et al. Preliminary observations from an open phase II trial of cannabidiol in children with autism spectrum disorder. In ACNP 61st Annual Meeting: Poster Abstracts P1 - P270. Neuropsychopharmacology. 2022;47(Supl 1):63-219. doi:10.1038/s41386-022-01484-1 3. Hacohen M, Stolar OE, Berkovitch M, Elkana O, Kohn E, Hazan A, Heyman E, Sobol Y, Waissengreen D, Gal E, Dinstein I. Children and adolescents with ASD treated with CBD-rich cannabis exhibit significant improvements particularly in social symptoms: an open label study. Transl Psychiatry. 2022;12(1):375. doi: 10.1038/s41398-022-02104-8. PMID: 36085294; PMCID: PMC9461457. 4. Heussler H, Duhig M, Hurst T, O’Neill C, Gutterman D, Palumbo JM, Sebree T. Longer term tolerability and efficacy of ZYN002 cannabidiol transdermal gel in children and adolescents with autism spectrum disorder (ASD): an open-label phase 2 study (BRIGHT [ZYN2-CL-030]). Adolescent Psychiatry (AACAP). 2020;12:24. 5. Heussler H, Fracp DM, Duhig M, Hurst T, O'Neill C, Gutterman D, Palumbo JM. [Bright (ZYN2-CL-030)] Tolerability and efficacy of ZYN002 cannabidiol transdermal gel in children and adolescents with autism spectrum disorder: an open-label phase 2 study. 2020 Virtual Meeting 2020 - AACAP. J Am Acad Child Adolesc Psychiatry. 2020;S264 – S265. doi: 10.1016/j.jaac.2020.08.463 6. Heussler H, Duhig M, Hurst T, O’Neill C, Agnese W, Palumbo J. Phase 2 BRIGHT (an Exploratory Open-Label Tolerability and Efficacy Study of ZYN002 Administered as a Transdermal Gel to Children and Adolescents With Autism Spectrum Disorder): Baseline Characteristics. J Neurotherapeutics. 2020;1321 – 1322. doi: 10.1007/s13311-020-00896-5 7. Lawson J, Conlon G, Cervantes P, Shalev R, Castellanos FX. 6.32 Assessing the effect of youth involvement in adverse event reporting during a clinical trial of cannabidiol for youth with autism spectrum disorder with complex verbal language. J Am Acad Child Adolesc Psychiatry. 2021;60(10):S168-9. 8. Palumbo JM, Heussler H, Duhig MJ, Hurst T, O’Neill C, Sebree T. Longer-term Tolerability and Efficacy of ZYN002 Cannabidiol Transdermal Gel in Children and Adolescents with Autism Spectrum Disorder: An Open-label Phase 2 Study (BRIGHT [ZYN2-CL-030]). Pediatrics. 2022;149. |
| **Wrong population** |
| 1. Pretzsch CM, Voinescu B, Lythgoe D, Horder J, Mendez MA, Wichers R, Ajram L, Ivin G, Heasman M, Edden RAE, Williams S, Murphy DGM, Daly E, McAlonan GM. Effects of cannabidivarin (CBDV) on brain excitation and inhibition systems in adults with and without Autism Spectrum Disorder (ASD): a single dose trial during magnetic resonance spectroscopy. Transl Psychiatry. 2019;9(1):313. doi: 10.1038/s41398-019-0654-8. PMID: 31748505; PMCID: PMC6868232. |
| **Wrong intervention** |
| 1. Khalaj M, Saghazadeh A, Shirazi E, Shalbafan MR, Alavi K, Shooshtari MH, Laksari FY, Hosseini M, Mohammadi MR, Akhondzadeh S. Palmitoylethanolamide as adjunctive therapy for autism: Efficacy and safety results from a randomized controlled trial. J Psychiatr Res. 2018;103:104-11. |

Supplementary Table S3. Main characteristics of the ongoing included randomized controlled trials

|  | **Registration number** | **Title** | **Population** | **Intervention** | **Comparator** | **Primary outcomes** | **Funding source** |
| --- | --- | --- | --- | --- | --- | --- | --- |
| **1** | [ACTRN12622001398796](https://anzctr.org.au/Trial/Registration/TrialReview.aspx?ACTRN=12622001398796) | *A Phase II/III Double-Blind, Randomised and Controlled-to-Open-Label Study Assessing the Efficacy of Full-Spectrum Medicinal Cannabis Plant Extract 0.08% THC (NTI164) on the Severity of Autism Spectrum Disorder in Young People.* | Participants between 8 and 17 years with level 2 or 3 ASD according to ADOS-2 criteria | Full-spectrum medicinal cannabis plant extract with 0.08% THC (NTI164) administered orally    8 weeks | Placebo:  virgin olive oil with a similar appearance to the active treatment | - Change in CGI-S from baseline to end of treatment | Neurotech International Limited |
| **2** | [NCT03202303](https://clinicaltrials.gov/study/NCT03202303) | *Cannabidivarin (CBDV) vs. Placebo in Children With Autism Spectrum Disorder (ASD).* | Participants between 5 and 18 years diagnosed with ASD according to ADOS-2 criteria | Cannabidivarin (CBDV) weight-based dosing of 10 mg/kg/day    12 weeks | Placebo: matched placebo weight-based dosing of 10 mg/kg/day | - Change in ABC-I from baseline to end of treatment | Montefiore Medical Center |
| **3** | [NCT04517799](https://clinicaltrials.gov/study/NCT04517799) | *A Double-Blind, Crossover Trial of Cannabidiol to Treat Severe Behavior Problems in Children With Autism.* | Boys between 7 and 14 years with severe ASD with substantial behavioral problems according to ADOS criteria | Oral purified CBD solution (EPIDIOLEX, purity of over 98%): 100 mg/mL solution, including sweetener to make it palatable.  Week 1: 5 mg/kg/day, divided into 2 doses  Week 2: 10 mg/kg/day, divided into 2 doses  Weeks 3 to 8: 20 mg/kg/day, divided into 2 doses.    8 weeks | Placebo: solution identical in color and taste, administered in the same volume as the CBD, and also weight dependent | - Change in RBS-R score from baseline to end of treatment - Change in CBCL score from baseline to end of treatment - Change in ADOS-2 score from baseline to end of treatment | University of California, San Diego |
| **4** | [NCT04520685](https://clinicaltrials.gov/study/NCT04520685) | *CASCADE: Cannabidiol Study in Children With Autism Spectrum Disorder (CASCADE)* | Participants between 5 and 17 years with moderate or severe ASD according to a medical or psychological professional | Oral cannabidiol 100mg/mL  Group 1: CBD titration dose 5mg/kg/day and the treatment dose will be 10mg/kg/day for 12 weeks.  Group 3: CBD titration dose 5mg/kg/day and the treatment dose will be 10mg/kg/day  27 weeks | Placebo: not specified.  15 weeks | - Change in ABC-2 score from baseline to end of treatment (irritability subscale) | University of Colorado, Denver |
| **5** | [NCT05015439](https://clinicaltrials.gov/study/NCT05015439?tab=table) / IRB00267739 | *Crossover Trial of Cannabidiol (CBD) Versus Placebo for Psychiatric Presentations in Adults With Autism Spectrum Disorder.* | Participants over 18 years with ASD according to DSM-5 | Cannabidiol: participants will receive cannabidiol, starting at 100 mg twice daily, and increased to 200 mg twice daily by week 3    6 weeks | Placebo: not specified. | - Change in ABC score (aberrant behaviors) | Johns Hopkins University |
| **6** | [NCT05626959](https://clinicaltrials.gov/study/NCT05626959) | *Evaluating the Efficacy of NTI164 in Young People With Autism Spectrum Disorder.* | Participants between 8 and 17 years with level 2 or 3 ASD according to ADOS-2 criteria | Full-Spectrum Medicinal Cannabis Plant Extract with less than 0.08% THC (NTI164) oil based: baseline dose of 5mg/kg/day increased weekly by 5mg/kg for a period of 4 weeks until the maximum tolerated dose or 20/mg/kg/day is achieved  8 weeks | Placebo: baseline dose of 5mg/kg/day increased weekly by 5mg/kg for a period of 4 weeks until the maximum tolerated dose or 20/mg/kg/day is achieved. | - Change in CGI-S | Fenix Innovation Group |
| **7** | NCT06526208 | *Investigating a Marijuana-based Compound as a Treatment for Anxiety in Autistic Adults* | Participants between 18 and 45 years old with ASD according to ADOS-2 criteria | MB-IMP, oil-based solution that will contain CBD and THC (23:1 ratio, respectively), to be taken orally.  Dosing will start at 0.25 ml per day (50 mg of CBD and 2.18 mg of THC) and will gradually increase to 1.0 ml per day (200 mg of CBD and 8.7 mg of TCH) by week 4.  Weeks 5 through 8 will be at the maximum dose, 2 ml per day (400 mg of CBD and 17.4 mg of THC). | Placebo: Medium Chain Triglyceride (MCT) oil. Does not contain CBD or THC | - Change in CGI-S - Change in socialization - Change in repetitive behaviors from baseline to the end of treatment - Change in a variety of aberrant behaviors from baseline to end of treatment | Southwest Autism Research & Resource Center |

ABC, Autistic Behavior Checklist; ADOS-2, Autism Diagnostic Observation Schedule-2; ASD, Autism Spectrum Disorder; CBCL, Child Behavior Checklist; CBD, Cannabidiol; CBVD, Cannabidivarin; CGI-I, Clinical Global Impressions scale - Global Improvement; CGI-S, Clinical Global Impressions scale - Severity of illness; DSM, Diagnostic and Statistical Manual of Mental Disorders; HSQ-ASD, Home Situations Questionnaire-Autism Spectrum Disorder; IQ, intelligence quotient; MCT, Medium Chain Triglycerides; RBS-R, Repetitive Behaviors Scale – Revised; THC, tetrahydrocanabinol; VABS-3, Vineland Adaptive Behavior Scales-3.

Supplementary Table S4: Cochrane Risk of Bias tool for all outcomes

| **Study/ domain** | | **Aran et al., 2021** | **NCT04745026** | **Parrella et al., 2024** | **Silva Junior et al., 2024** |
| --- | --- | --- | --- | --- | --- |
| **Random sequence generation (selection bias)** | | **LOW RISK**  Quote: *"Randomization scheme was generated by BioStats Statistical Consulting Ltd. (an external biostatistical consulting firm). The blocked randomization was performed using SAS statistical software V9.4 via the plan procedure with a fixed block size. The randomization scheme and lists were sent to the center for both periods of the study. The randomization list included sequential numbers and an assigned 3-letter code (several codes were assigned for each of the 3 treatment options). Randomization was kept at 1:1:1 ratio. The randomization list was encrypted by password and was sent prior to first dosing to the principal investigator.*" (supplementary file 1)    Comment: randomization method is considered adequate. | **LOW RISK**  Quote: *“Participants will be stratified based on their age (6 to 11 years old, 12 to 17 years old), use of antipsychotics (on versus off), and region (North America versus Rest of the World) and will be randomized to receive (...)”*  Comment: although no details were given of the software used to generate the random sequence assignment list, it is likely that it was used, since three different characteristics were stratified. | **LOW RISK**  Quote: *“Participants were randomly assigned (via block randomisation) to receive either Medigrowth CBD100 or placebo oil during each intervention period of the trial.”*    Comment: although no details were given of the software used to generate the random sequence assignment list, it is likely that it was used, since a block randomization was applied. | **LOW RISK**  Quote: *“The randomization was done using the True Random Number Service, available at:* [*www.random.org*](https://www.random.org/)*. All the researchers who had direct contact with the patients were blinded to the treatment provided, except the pharmacy student, who was assigned with delivering the vials, weekly, to the researching physician. Finally, baseline evaluations, performed by the same child and adolescent psychiatrist responsible for this reseach, were scheduled with all children participating in this study, to whom the products for the test were also delivered.”*    Comment: randomization method is considered adequate. |
| **Allocation concealment (selection bias)** | | **LOW RISK**  Quote: *"The randomization scheme and lists were sent to the center for both periods of the study. The randomization list included sequential numbers and an assigned 3-letter code (several codes were assigned for each of the 3 treatment options). Randomization was kept at 1:1:1 ratio. The randomization list was encrypted by password and was sent prior to first dosing to the principal investigator."* (supplementary file 1)    Comment: allocation concealment method is considered adequate. | **LOW RISK**  Quote: *“Participants will be stratified based on their age (6 to 11 years old, 12 to 17 years old), use of antipsychotics (on versus off), and region (North America versus Rest of the World) and will be randomized to receive (...)”*  Comment: although the strategy used to maintain allocation concealment has not been clearly reported, it is possible that it happened, since a software was most likely used to generate the randomization sequence. | **LOW RISK**  Quote: *“Participants were randomly assigned (via block randomisation) to receive either Medigrowth CBD100 or placebo oil during each intervention period of the trial.”*    Comment: although the strategy used to maintain allocation concealment has not been clearly reported, it is possible that it happened, since a block randomization was applied. | **LOW RISK**  Quote: *“The randomization was done using the True Random Number Service, available at:* [*www.random.org*](https://www.random.org/)*. All the researchers who had direct contact with the patients were blinded to the treatment provided, except the pharmacy student, who was assigned with delivering the vials, weekly, to the researching physician. Finally, baseline evaluations, performed by the same child and adolescent psychiatrist responsible for this reseach, were scheduled with all children participating in this study, to whom the products for the test were also delivered.”*    Comment: allocation concealment method is considered adequate. |
| **Blinding of participants and personnel (Performance bias)** | **Global assessment of symptoms** | **HIGH RISK**  Quote: *"The study whole-plant extract, the pure cannabinoids as well as placebo were packed in similar bottles and had similar color, taste and smell characteristics. The treatment products and placebo were both dissolved in olive oil. Addition of paprika color and oleoresin oregano to the placebo solution disguised the color completely. Addition of oleoresin oregano at high concentration was applied to mask the tastes of CBD and THC and raspberry flavor was added to mask the aroma (confirmed by a group of experienced tasters).*  *The code key was kept by BioStats Statistical Consulting Ltd. until study end. Neither the principal investigator nor any other team member or individual had access to the codes until study end. No unblinding occurred during the study."* (supplementary file)    Comment: Although the study used a strategy to blind participants and personnel, the use of gradual increase of dosage over a short period of time increases the likelihood of breaking the blinding due to the occurrence of adverse events that were frequent among the groups and are related to the intervention. | **HIGH RISK**  Quote: *“Patients will be administered 5 mg/kg/day GWP42003-P or matching volumes of placebo for 1 week and then 10 mg/kg/day GWP42003-P or matching volumes of placebo for 11 weeks. At the end of treatment, patients will taper the medication over 1 week.”*  *“Patients who were randomized to placebo for 12 weeks.”*  Comment: Although the study used a strategy to blind participants and personnel, the use of gradual increase of dosage over a short period of time in only one group increases the likelihood of breaking the blinding. | Outcome not assessed by the study. | **HIGH RISK**  Quote: *"The CBD-rich cannabis extract and the product without it had the same consistency, color, odor, and other organoleptic characteristics, making it impossible for patients and the multidisciplinary team accompanying them to discern between the two."*    Comment: Although the study used a strategy to blind participants and personnel, the use of gradual increase of dosage over a short period of time increases the likelihood of breaking the blinding due to the occurrence of adverse events that were frequent among the groups and are related to the intervention. |
|  | **Symptom severity** | **HIGH RISK**  *Idem previous* | **HIGH RISK**  *Idem previous* | **HIGH RISK**  Quote: *“In addition, the placebo for the CBD100 medication consisted of MCT as an excipient, matched to CBD100 in taste to maintain blinding integrity throughout the trial.(…) Medigrowth CBD100 was administered orally in weight-based doses calculated by the investigator. In each intervention period, the starting dose was 5mg/kg body weight/day. The dose was increased to 10 mg/kg/day by the second week, with parents or guardians trained to facilitate sublingual administration during the first visit. Placebo oil was administered following the same protocol.”*    Comment: Although the study used a strategy to blind participants, the use of gradual increase of dosage over a short period of time increases the likelihood of breaking the blinding due to the occurrence of adverse events that were frequent among the groups and are related to the intervention. | **HIGH RISK**  *Idem previous* |
|  | **Serious adverse events** | **HIGH RISK**  *Idem previous* | **HIGH RISK**  *Idem previous* | Outcome not assessed by the study. | **HIGH RISK**  *Idem previous* |
|  | **Quality of life** | Outcome not assessed by the study. | Outcome not assessed by the study. | Outcome not assessed by the study. | Outcome not assessed by the study. |
|  | **Any adverse event** | **HIGH RISK**  *Idem previous* | **HIGH RISK**  *Idem previous* | **HIGH RISK**  *Idem previous* | **HIGH RISK**  *Idem previous* |
|  | **Adaptive behavior** | **HIGH RISK**  *Idem previous* | **HIGH RISK**  *Idem previous* | **HIGH RISK**  *Idem previous* | Outcome not assessed by the study. |
| **Blinding of outcome assessment (detection bias)** | **Global assessment of symptoms** | **LOW RISK**  Quote: *"Neither the principal investigator nor any other team member or individual had access to the codes until study end. No unblinding occurred during the study."*    Comment: although the assessment of the tool can be considered subjective, the same outcome assessor blinded to the participants' allocation evaluated everyone at the start of the study and after 12 weeks. | **HIGH RISK**  Quote: *“The CGI-I is a 7-point scale that requires the clinician to assess how much the patient's illness has improved or worsened relative to a baseline state at the beginning of the intervention. The clinician is asked: Compared to the patient's condition at admission to the project, how much has the patient changed?”*  Comment: Although the protocol reports outcome assessors as blinded, this is a subjective outcome that depends on the previous and the final clinician’s opinion about the participant condition. | Outcome not assessed by the study. | **HIGH RISK**  Quote: *"whether the caregiver was in doubt, did not notice or could not see improvement with the test product, at the final consultation, before researcher and participants knew if the child was in the treated or placebo group."* (publication)    Comment: ATEC-T and CARS were assessed through a semi-structured interview between the attending psychiatrist and the family member or career. |
|  | **Symptom severity** | **LOW RISK**  *Idem previous* | **HIGH RISK**  Quote: *“The caregiver-assessed ABC was designed to assess the presence and severity of various problem behaviors commonly observed in individuals diagnosed with intellectual and developmental disability.”*  Comment: Although the protocol reports outcome assessors as blinded, this is a caregiver-assessed highly subjective outcome that depends on the opinion of the caregiver, the same person that provides the intervention for the participant. | **HIGH RISK**  Quote: *“During the testing visits, parents or guardians of participants were required to complete behavioural questionnaires”*    Comment: SRS-2 was assessed by parents/caregivers through a paper-based interview. | **HIGH RISK**  *Idem previous* |
|  | **Serious adverse events** | **LOW RISK**  Quote: *"Neither the principal investigator nor any other team member or individual had access to the codes until study end. No unblinding occurred during the study."*    Comment: apparently the outcome assessor was blinded to the participants allocation and apparently the counting method was used in all 3 groups. | **HIGH RISK**  Quote: *“Measure Type: Count of Participants”*  Comment: The caregiver is the person that provides the intervention and follows the participant throughout the study, witnessing possible adverse events. | Outcome not assessed by the study. | **HIGH RISK**  Quote: *"whether the caregiver was in doubt, did not notice or could not see improvement with the test product, at the final consultation, before researcher and participants knew if the child was in the treated or placebo group."* (publication)    Comment: it is possible that the outcome counting was affected by the perception of the parents or careers. |
|  | **Quality of life** | Outcome not assessed by the study. | Outcome not assessed by the study. | Outcome not assessed by the study. | Outcome not assessed by the study. |
|  | **Any adverse event** | **LOW RISK**  *Idem previous* | **HIGH RISK**  *Idem previous* | **HIGH RISK**  Quote: *“Parents or guardians of participants provided ASD diagnostic reports, completed behavioural questionnaires, and administered the study intervention daily.”*    Comment: it is possible that the outcome counting was affected by the perception of the parents or careers. | **HIGH RISK**  *Idem previous* |
|  | **Adaptive behavior** | **LOW RISK**  Quote: *"Neither the principal investigator nor any other team member or individual had access to the codes until study end. No unblinding occurred during the study."*    Comment: apparently the outcome assessor was blinded to the participants’ allocation. Although the assessment of the tool can be considered subjective, the same assessor blinded to the participants' allocation evaluated everyone at the start of the study and after 12 weeks. | **HIGH RISK**  Quote: *"The VABS-3 scales assess what a person does, rather than what he or she can do. The Vineland-3 assesses adaptive behavior in 3 domains: Communication, Daily Living Skills, and Socialization. Each domain is comprised of 3 subdomains: receptive expression and written (communication); personal, domestic and community (daily living skills); Interpersonal relationships, play and leisure and copying skills (socialization).”*  Comment: outcome assessment is subjective through a semi-structured interview with the caregivers. | **HIGH RISK**  Quote: *“During the testing visits, parents or guardians of participants were required to complete behavioural questionnaires”*    Comment: outcome was assessed by parents/caregivers through a paper-based interview. | Outcome not assessed by the study. |
| **Incomplete outcome data (attrition bias)** | **Global assessment of symptoms** | **LOW RISK**  Comment: according to figure 3, around 91.3% of the randomized participants contributed with data for this outcome and the losses appear to be balanced between the groups. | **HIGH RISK**  Comment: 72 (70%) of the 103 randomized participants contributed with data for this outcome. | Outcome not assessed by the study. | **LOW RISK**  Quote: *"During recruitment and laboratory testing prior to the start of the clinical trial, four children dropped out of the study, three were recruited for the control group and one for the cannabis group, thus resulting in a final sample of 60 children (31 in the treatment group and 29 in the placebo group). The reason was that they did not live in the city where the clinical trial was executed and had difficulty travelling there."*    Comment: 64 children were randomized and, according to table 3, 60 (93.7%) contributed with data. |
|  | **Symptom severity** | **HIGH RISK**  Comment: 150 participants were randomized, but only 98 (65.33%) provided data for this outcome. It is not possible to state that missing data would not alter the results of this outcome. | **HIGH RISK**  Comment: 91 (88%) of the 103 randomized participants contributed with data for this outcome. Losses were unbalanced between the groups, with 9/49 in the intervention group and 3/54 in the control group. | **HIGH RISK**  Comment: 34 participants were randomized, but 7 (20.5%) were lost to follow up.  Although both groups lost a balanced number of participants and only 1 of them may have been directly related to the intervention, considering the overall scenario of the study, it is not possible to state that missing this data would not alter the results of this outcome. | **LOW RISK**  *Idem previous* |
|  | **Serious adverse events** | **LOW RISK**  Comment: according to Table 2 from additional file, the data presented include participants from both phases of the crossover and 282 participants contributed with data for this outcome representing a lost to follow up of 18 (6%), balanced between groups. It is possible that missing data would not alter the results of this outcome. | **LOW RISK**  Comment: all participants contributed with data for this outcome. | Outcome not assessed by the study. | **UNCLEAR**  Quote: *“CBD-rich cannabis extract was found to improve one of the diagnostic criteria for ASD (social interaction), as well as often co-existing features, and to have few and no serious adverse effects.”*  Comment: not enough information to judge. |
|  | **Quality of life** | Outcome not assessed by the study. | Outcome not assessed by the study. | Outcome not assessed by the study. | Outcome not assessed by the study. |
|  | **Any adverse event** | **LOW RISK**  Comment: considering that the data presented includes participants from both phases of the crossover, it seems that all participants randomized in both phases contributed with data. | **LOW RISK**  *Idem previous* | **HIGH RISK**  Comment: 34 participants were randomized, but 7 (20.5%) were lost to follow up.  Although both groups lost a balanced number of participants and only 1 of them may have been directly related to the intervention, considering the overall scenario of the study, the loss of these participants could have a major impact on the final effect of the intervention. | **LOW RISK**  Comment: according to table 2, 64 children were randomized and 60 (93.7%) contributed with data. |
|  | **Adaptive behavior** | **HIGH RISK**  Comment: according to table 4, around 80.6% of the randomized participants contributed with data for this outcome, but the losses were balanced between the 3 groups (10/50 cannabis extract group, 8/50 pure cannabis group, 11/50 placebo group). It is not possible to state that the missing data would not alter the result. | **HIGH RISK**  Comment: 82 (79%) of the 103 randomized participants contributed with data for this outcome. | **HIGH RISK**  Comment: 34 participants were randomized, but 7 (20.5%) were lost to follow up.  Although both groups lost a balanced number of participants and only 1 of them may have been directly related to the intervention, considering the overall scenario of the study, the loss of these participants could have a major impact on the final effect of the intervention. | Outcome not assessed by the study. |
| **Selective reporting (reporting bias)** | | **HIGH RISK**  Comment: according to protocol NCT02956226, 3 follow-up time-point measurements were planned (4, 8 and 12 weeks), but only the difference between baseline and week 12 was presented.  In addition, during the study global assessment of symptoms changed from secondary to primary after a second tool was included, because the previously selected tool (HSQ-ASD) was considered insufficient to identify intended differences for the participants by the researchers. | **LOW RISK**  Comment: all outcomes and time points assessments planned at the prospective protocol (NCT04745026) had their results reported. | **LOW RISK**  Comment: protocol ACTRN12622000437763 prospectively registered, planned outcomes and time-points reported in the article. | **HIGH RISK**  Quote: *"Date of first enrolment: 02/12/2019. Date of registration: 17/12/2020. Prospective registration: No." (protocol RBR-5wr2cqq)*    Comment: it is not possible to confirm whether the outcome data was assessed as planned, as the protocol was registered retrospectively. |
| **Other bias** | | **LOW RISK**  Comment: no other risk of bias was detected. | **LOW RISK**  Comment: no other risk of bias was detected. | **LOW RISK**  Comment: no other risk of bias was detected. | **LOW RISK**  Comment: no other risk of bias was detected. |

ASD, Autism Spectrum Disorder; ATEC-T, Autism Treatment Evaluation Checklist; CARS, Childhood Autism Rating Scale; CBD, canabidiol; CGI-I, Clinical Global Impressions – Improvement; HSQ-ASD, Home Situations Questionnaire-ASD; LEAP, Liverpool Adverse Events Profile; RR, relative risk; SRS-2, Social Responsiveness Scale - 2ª; THC, Tetrahydrocanabidiol.

Supplementary Table S5. Summary of findings tables

1. Summary of findings table of whole cannabis extract versus placebo for autism

| Cannabis extract (whole plant) compared to placebo for Autism Spectrum Disorder (ASD) | | | | | | |
| --- | --- | --- | --- | --- | --- | --- |
| **Patient or population:** Children and adolescents (between 5 and 21 years) with ASD.  **Setting:** Ambulatory / home care.  **Intervention:** Whole plant extraction of Cannabis dissolved in olive oil (167 mg/ml CBD and 8.35 mg/ml THC).  **Comparison:** Placebo. | | | | | | |
| Outcomes | **Anticipated absolute effects^*^** (95% CI) | | Relative effect  (95% CI) | № of participants  (studies) | Certainty of the evidence  (GRADE) | Comments |
|  | Risk with placebo | Risk with cannabis |  |  |  |  |
| Global assessment of symptoms  (CGI-I)  12 weeks | 213 per 1.000 | **487 per 1.000**  (260 to 913) | **RR 2.30**  (1.23 to 4.30) | 92  (1 RCT) | ⨁⨁◯◯  Low^a^ | Cannabis whole plant may increase the risk of improvement on the CGI scale. |
| Symptom severity  (SRS-2)  12 weeks | - | **-** | **MD –11.0**  (-24.47 to 2.47) | 65  (1 RCT) | ⨁◯◯◯  Very low^a,b^ | The effect of cannabis whole plant on symptom severity is uncertain. |
| Serious adverse events  12 weeks | 11 per 1.000 | **21 per 1.000**  (2 to 228) | **RR 1.09**  (0.07 to 16.90) | 94  (1 RCT) | ⨁◯◯◯  Very low^a,c^ | The effect of cannabis whole plant on the incidence of serious adverse events is uncertain. |
| Quality of life | - | **-** | **-** | - | - | Outcome not assessed by the study. |
| Any adverse event  12 weeks | 939 per 1.000 | **986 per 1.000**  (873 to 49) | **RR 1.06**  (0.93 to 1.21) | 96 (1 RCT) | ⨁◯◯◯  Very low^a,c^ | The effect of cannabis whole plant on the incidence of any adverse event is uncertain. |
| Adaptive behavior  (HSQ-ASD)  12 weeks | - | - | **MD –0.43**  (-1.20 to 0.34) | 79  (1 RCT) | ⨁◯◯◯  Very low^a,b^ | The effect of cannabis whole plant on adaptative behavior is uncertain. |
| ***The risk in the intervention group** (and its 95% confidence interval) is based on the assumed risk in the comparison group and the **relative effect** of the intervention (and its 95% CI).  **CI:** confidence interval; **MD:** mean difference; **RR:** risk ratio | | | | | | |
| **GRADE Working Group grades of evidence**  **High certainty:** we are very confident that the true effect lies close to that of the estimate of the effect.  **Moderate certainty:** we are moderately confident in the effect estimate: the true effect is likely to be close to the estimate of the effect, but there is a possibility that it is substantially different.  **Low certainty:** our confidence in the effect estimate is limited: the true effect may be substantially different from the estimate of the effect.  **Very low certainty:** we have very little confidence in the effect estimate: the true effect is likely to be substantially different from the estimate of effect. | | | | | | |
| 1. **Risk of bias:** high risk of bias for the domains 'blinding of participants and personnel' and 'selective reporting' (reduction by two levels). 2. **Risk of bias:** high risk of bias for the ‘incomplete outcome data’ (reduction by one level). 3. **Imprecision**: result consistent with a substantial decrease or increase in the event rate, probably due to the low rate of events (reduction by two levels). | | | | | | |

1. Summary of findings table of purified cannabis extract versus placebo for autism.

| Cannabis extract (purified) compared to placebo for Autism Spectrum Disorder (ASD) | | | | | | |
| --- | --- | --- | --- | --- | --- | --- |
| **Patient or population:** Children and adolescents (between 5 and 21 years) with ASD.  **Setting:** Ambulatory / home care.  **Intervention:** Purified Cannabis extraction (99% pure CBD) dissolved in olive oil (167 mg/ml CBD and 8.35 mg/ml THC).  **Comparison:** Placebo. | | | | | | |
| Outcomes | **Anticipated absolute effects^*^** (95% CI) | | Relative effect  (95% CI) | № of participants  (studies) | Certainty of the evidence  (GRADE) | Comments |
|  | Risk with placebo | Risk with cannabis |  |  |  |  |
| Global assessment of symptoms  (CGI-I)  12 weeks | 213 per 1.000 | **377 per 1.000**  (194 to 734) | **RR 1.78**  (0.91 to 3.45) | 92  (1 RCT) | ⨁◯◯◯  Very low^a,b^ | The effects of the purified cannabis on the global assessment of symptoms is uncertain. |
| Symptom severity  (SRS-2)  12 weeks | - | **-** | **MD –15.09**  (-27.46 to -2.54) | 69  (1 RCT) | ⨁◯◯◯  Very low^a,c^ | The effects of the purified cannabis on the symptom severity is uncertain. |
| Serious adverse events  12 weeks | 11 per 1.000 | **32 per 1.000**  (3 to 304) | **RR 2.09**  (0.20 to 22.24) | 96  (1 RCT) | ⨁◯◯◯  Very low^a,b^ | The effects of the purified cannabis on the incidence of serious adverse events is uncertain. |
| Quality of life | - | **-** | **-** | - | - | Outcome not assessed by the study. |
| Any adverse event  12 weeks | 898 per 1.000 | **1.000 per 1.000**  (817 to 1.000) | **RR 1.09**  (0.97 to 1.23) | 98  (1 RCT) | ⨁◯◯◯  Very low^a,b^ | The effects of the purified cannabis on the incidence of any adverse events is uncertain. |
| Adaptive behavior  (HSQ-ASD)  12 weeks | - | - | **MD –0.40**  (-1.24 to 0.44) | 81  (1 RCT) | ⨁◯◯◯  Very low^a,c^ | The effects of the purified cannabis on the adaptative behavior is uncertain. |
| ***The risk in the intervention group** (and its 95% confidence interval) is based on the assumed risk in the comparison group and the **relative effect** of the intervention (and its 95% CI).  **CI:** confidence interval; **MD**: mean difference; **RR:** risk ratio. | | | | | | |
| **GRADE Working Group grades of evidence**  **High certainty:** we are very confident that the true effect lies close to that of the estimate of the effect.  **Moderate certainty:** we are moderately confident in the effect estimate: the true effect is likely to be close to the estimate of the effect, but there is a possibility that it is substantially different.  **Low certainty:** our confidence in the effect estimate is limited: the true effect may be substantially different from the estimate of the effect.  **Very low certainty:** we have very little confidence in the effect estimate: the true effect is likely to be substantially different from the estimate of effect. | | | | | | |
| 1. **Risk of bias:** high risk of bias for the domain 'blinding of participants e personnel' and 'selective reporting' (reduction by two levels). 2. **Imprecision**: result consistent with a decrease or substantial increase in the event rate (reduction by two levels). 3. **Risk of bias**: high risk of bias for the domain ‘incomplete outcome data’ (reduction by one level). | | | | | | |

3. Summary of findings table of CBD oil (GWP42003-P) versus placebo for autism.

| CBD oil (GWP42003-P) compared to placebo for Autism Spectrum Disorder (ASD) | | | | | | |
| --- | --- | --- | --- | --- | --- | --- |
| **Patient or population:** Children and adolescents (between 6 and 17 years) with ASD.  **Setting:** Ambulatory / home care.  **Intervention:** 5 mg/kg/day GWP42003-P for 1 week and then 10 mg/kg/day GWP42003 oral solution (100 mg/mL CBD in sesame oil with anhydrous ethanol, ethanol 10% v/v, sweetener [sucralose].  **Comparison:** Placebo (matching oral solution containing sesame oil with anhydrous ethanol, sweetener (sucralose), strawberry flavoring, and beta carotene). | | | | | | |
| Outcomes | **Anticipated absolute effects^*^** (95% CI) | | Relative effect  (95% CI) | № of participants  (studies) | Certainty of the evidence  (GRADE) | Comments |
|  | Risk with placebo | Risk with CBD oil (GWP42003-P) |  |  |  |  |
| Global assessment of symptoms  (CGI-I)  12 weeks | 316 per 1.000 | **354 per 1.000**  (183 to 679) | **RR 1.12**  (0.58 to 2.15) | 72  (1 RCT) | ⨁◯◯◯  Very low^a,b^ | The effect of GWP42003-P on the global assessment of symptoms is uncertain. |
| Symptom severity  (SRS-2)  12 weeks | - | - | - | - | - | Data for this outcome could not be assessed, as it was presented according to sub-scales. The total score was not presented. |
| Serious adverse events  12 weeks | 19 per 1.000 | **41 per 1.000**  (4 to 436) | **RR 2.20**  (0.21 to 23.56) | 103  (1 RCT) | ⨁◯◯◯  Very low^b,c^ | The effects of the GWP42003-P on the incidence of serious adverse events is uncertain. |
| Quality of life | - | **-** | **-** | - | - | Outcome not assessed by the study. |
| Any adverse event  12 weeks | - | - | - | - | - | Data for this outcome could not be assessed due to lack of clear data about the number/proportion of participants with any adverse event. |
| Adaptive behavior  (VABS-3 ABC)  12 weeks | - | **-** | **MD -0.7**  (-5.46 to 4.06) | 63  (1 RCT) | ⨁◯◯◯  Very low^a,b^ | The effect of GWP42003-P on the adaptative behavior is uncertain. |
| ***The risk in the intervention group** (and its 95% confidence interval) is based on the assumed risk in the comparison group and the **relative effect** of the intervention (and its 95% CI).  **CI:** confidence interval; **MD:** mean difference; **RR:** risk ratio. | | | | | | |
| **GRADE Working Group grades of evidence**  **High certainty:** we are very confident that the true effect lies close to that of the estimate of the effect.  **Moderate certainty:** we are moderately confident in the effect estimate: the true effect is likely to be close to the estimate of the effect, but there is a possibility that it is substantially different.  **Low certainty:** our confidence in the effect estimate is limited: the true effect may be substantially different from the estimate of the effect.  **Very low certainty:** we have very little confidence in the effect estimate: the true effect is likely to be substantially different from the estimate of effect. | | | | | | |
| 1. **Risk of bias:** high risk of bias for the domain 'blinding of participants and personnel', 'blinding of outcome assessors' and ‘incomplete outcome data’ (reduction by two levels). 2. **Imprecision**: result consistent with a substantial decrease or increase in the event rate (reduction by two levels). 3. **Risk of bias:** high risk of bias for the domain 'blinding of participants and personnel' and 'blinding of outcome assessors' (reduction by two levels). | | | | | | |

4.  Summary of findings table of cannabis oil (Medigrowth CBD100 oil) versus placebo for autism.

| Cannabis oil (Medigrowth CBD100 oil) compared to placebo for Autism Spectrum Disorder (ASD) | | | | | | |
| --- | --- | --- | --- | --- | --- | --- |
| **Patient or population:** Children (between 5 and 12 years) with ASD.  **Setting:** Ambulatory / home care.  **Intervention:** Weight-based dosing of 10 mg/kg/day of cannabidiol (Medigrowth CBD100) for 12 weeks (delivered sublingually via syringe dispenser).  **Comparison:** Placebo (MCT (coconut) oil delivered sublingually from syringe dispenser) | | | | | | |
| Outcomes | **Anticipated absolute effects^*^** (95% CI) | | Relative effect  (95% CI) | № of participants  (studies) | Certainty of the evidence  (GRADE) | Comments |
|  | Risk with placebo | Risk with cannabis |  |  |  |  |
| Global assessment of symptoms  12 weeks | - | **-** | **-** | - | - | Outcome not assessed by the study. |
| Symptom severity  (SRS-2)  12 weeks | - | - | **MD -14.00**  (-39.94 to 11.94) | 27  (1 RCT) | ⨁◯◯◯  Very low^a,b^ | The effects of the cannabis oil on the symptom severity is uncertain. |
| Serious adverse events  12 weeks | - | - | **-** | - | - | The authors reported that they had not observed any serious adverse events. |
| Quality of life | - | **-** | **-** | - | - | Outcome not assessed by the study. |
| Any adverse event  12 weeks | 0 events per 14 participants | 2 events per 13 participants | - | 27  (1 RCT) | ⨁◯◯◯  Very low^a,c^ | The effects of the cannabis oil on the incidence of any adverse event is uncertain. |
| Adaptive behavior  (VABS-3)  12 weeks | - | - | **MD 2.60**  (-10.23 to 15.43) | 27  (1 RCT) | ⨁◯◯◯  Very low^a,b^ | The effects of the cannabis oil on the adaptative behavior is uncertain. |
| ***The risk in the intervention group** (and its 95% confidence interval) is based on the assumed risk in the comparison group and the **relative effect** of the intervention (and its 95% CI).  **CI:** confidence interval; **MD:** mean difference; **RR:** risk ratio. | | | | | | |
| **GRADE Working Group grades of evidence**  **High certainty:** we are very confident that the true effect lies close to that of the estimate of the effect.  **Moderate certainty:** we are moderately confident in the effect estimate: the true effect is likely to be close to the estimate of the effect, but there is a possibility that it is substantially different.  **Low certainty:** our confidence in the effect estimate is limited: the true effect may be substantially different from the estimate of the effect.  **Very low certainty:** we have very little confidence in the effect estimate: the true effect is likely to be substantially different from the estimate of effect. | | | | | | |
| 1. **Risk of bias:** high risk of bias for the domains 'blinding of participants and personnel', ‘blinding of outcome assessors’ and 'incomplete outcome data' (reduction by two levels). 2. **Imprecision:** result consistent with a substantial decrease or increase in the assessment of the score (reduction by one level). 3. **Imprecision**: results for this outcome were not calculated due to very low number of events (reduction by two levels). | | | | | | |

5. Summary of findings table of cannabis extraction (CBD 0.5%) versus placebo for autism.

| Cannabis extraction (CBD 0.5%) compared to placebo for Autism Spectrum Disorder (ASD) | | | | | | |
| --- | --- | --- | --- | --- | --- | --- |
| **Patient or population:** Children (between 5 and 12 years) with ASD.  **Setting:** Ambulatory / home care.  **Intervention:** Cannabis extraction with CBD 0.5% (5 mg/mL), with a THC and CBD proportion of 9:1.  **Comparison:** Placebo. | | | | | | |
| Outcomes | **Anticipated absolute effects^*^** (95% CI) | | Relative effect  (95% CI) | № of participants  (studies) | Certainty of the evidence  (GRADE) | Comments |
|  | Risk with placebo | Risk with cannabis |  |  |  |  |
| Global assessment of symptoms  (ATEC-T)  12 weeks | - | **-** | **-** | - | - | Data for this outcome could not be assessed due to inconsistencies between publications of the study. |
| Symptom severity  (CARS)  12 weeks | - | **-** | **-** | - | - | Data for this outcome could not be assessed due to inconsistencies between publications of the study. |
| Serious adverse events  12 weeks | - | - | **-** | - | - | The authors reported that they had not observed any serious adverse events. |
| Quality of life | - | **-** | **-** | - | - | Outcome not assessed by the study. |
| Any adverse event  12 weeks | 172 per 1.000 | **129 per 1.000**  (38 to 434) | **RR 0.75**  (0.22 to 2.52) | 60  (1 RCT) | ⨁◯◯◯  Very low^a,b^ |  |
| Adaptive behavior | - | - | **-** | - | - | Outcome not assessed by the study. |
| ***The risk in the intervention group** (and its 95% confidence interval) is based on the assumed risk in the comparison group and the **relative effect** of the intervention (and its 95% CI).  **CI:** confidence interval; **MD:** mean difference; **RR:** risk ratio. | | | | | | |
| **GRADE Working Group grades of evidence**  **High certainty:** we are very confident that the true effect lies close to that of the estimate of the effect.  **Moderate certainty:** we are moderately confident in the effect estimate: the true effect is likely to be close to the estimate of the effect, but there is a possibility that it is substantially different.  **Low certainty:** our confidence in the effect estimate is limited: the true effect may be substantially different from the estimate of the effect.  **Very low certainty:** we have very little confidence in the effect estimate: the true effect is likely to be substantially different from the estimate of effect. | | | | | | |
| 1. **Risk of bias:** high risk of bias for the domains 'blinding of participants and personnel', ‘blinding of outcome assessors’ and 'selective reporting' (reduction by two levels). 2. **Imprecision**: result consistent with a decrease or substantial increase in the event rate (reduction by two levels). | | | | | | |
